# Rethinking the Evolution of Tubulin Polymerization Promoting Proteins

**DOI:** 10.3390/biology14121784

**Published:** 2025-12-14

**Authors:** Ferenc Orosz

**Affiliations:** Institute of Molecular Life Sciences, HUN-REN Research Centre for Natural Sciences, 1117 Budapest, Hungary; orosz.ferenc@ttk.hu

**Keywords:** p25alpha domain, Opisthokonta, Choanoflagellata, *Tunicaraptor*, Opimoda+, Diphoda+, Heterolobosea

## Abstract

TPPP (tubulin polymerization promoting protein)-like proteins are found throughout the living world. The individual members of this protein family are distinguished according to how many times and how completely their characteristic structural element, the p25alpha domain, is found in them. Phylogenomic occurrences of the members of the family differ from each other. Animals, fungi, algae, and various groups of unicellular organisms have their characteristic proteins. The two phylogenomic multi-supergroups, Opimoda+ and Diphoda+, show very different patterns in the occurrence of TPPP types. By using BLAST search in protein and nucleotide databases, we found that the previously known phylogenomic distribution is not strictly true, e.g., fungal type TPPPs are not only found in fungi. We primarily analyzed the Opisthokonta clade but also examined broader relationships. It was confirmed that the occurrence of TPPPs/genes is linked to the presence of the eukaryotic flagellum. A TPPP that contains the entire p25alpha domain twice and occurs only in Opisthokonta was identified. We also identified a TPPP in choanoflagellates and in the uncertainly classified Opisthokonta *Tunicaraptor unikontis*, which was previously known only in the Diphoda+ clade. On the other hand, we found an Opisthokonta (Opimoda+)-specific TPPP in a Heterolobosea (Diphoda+). Based on these results, we need to rethink the evolutionary history of TPPPs.

## Simple Summary

So-called TPPP-like proteins are found in most of the living world. The individual members of this protein family are distinguished according to how many times and how completely their characteristic structural element, the p25alpha domain, is found in them. Occurrences of individual members of the family are very different from each other. Animals, fungi, algae, and various groups of unicellular organisms have their characteristic proteins; plants do not have them. The article is about how the previously understood specific distribution of proteins and the genes that code for them is not strictly true, and what the reasons are for these (apparent) discrepancies. In addition, we have also identified a previously unknown type of protein. These results were achieved by analyzing protein and genetic databases. Some members of the protein family are of particular importance for health. In humans, they play a role in certain neurodegenerative diseases, such as Parkinson’s disease, in certain tumors, and in male fertility. Other members of the family play important roles in the pathogens that cause malaria and various illnesses in livestock. Therefore, the more we know about these related proteins, the more we can understand their role in specific diseases.

## 1. Introduction

**Figure 1:**
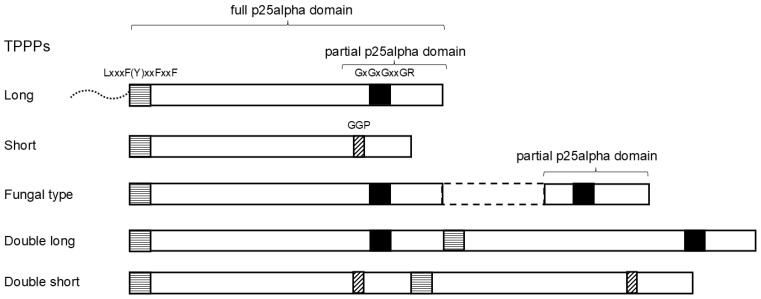
Schematic structures of various TPPPs. Squares and a rectangle indicate conservative sequences. Dotted line indicates an unstructured N-terminus present in some long TPPPs. Dashed lines indicate a sequence of a fungal type TPPP that does not belong to p25alpha domains.

The first representative and namesake of the TPPP family (tubulin polymerization promoting protein) was identified from a bovine brain and named p25 [1]. (For a recent review of TPPPs see [2].) It received its current name later, when it was characterized, also from a bovine brain, establishing its tubulin-polymerizing and microtubule-stabilizing effects [3]. The name ‘p25’ has also been retained: the domain characteristic of TPPPs, which encompasses most of their sequence, is called ‘p25alpha’ (Pfam05517 or IPR008907). It should be noted that the p25alpha domain is not found in TPPPs only; it is also present in other proteins in combination with other domains [4]. It was also shown that there are two main types of p25alpha domains, and thus TPPPs, a long and a short one [5]. In addition to the different lengths, the two types are characterized by distinctive sequential similarities and differences. The most typical is that the C-terminus of the long TPPP contains a highly conserved region of about 30–32 amino acids, which is absent in the short type [3]. Part of this conserved region is the GxGxGxxGR motif [3]. Specific sequences are also present in short TPPPs, e.g., a GGP triplet always occurs. A few other characteristic sequences are shown in Figure 1.

It was found that the long TPPP occurs in practically all animals; the only exception is the placozoan *Trichoplax adhaerens* [5]. (The complete genome of this has been known for a long time and, indeed, does not contain TPPP [6].) The long TPPP was also found in other early-branching Opisthokonta: in fungi that reproduce by zoospores equipped with a flagellum and in *Monosiga brevicollis* (Choanoflagellata) [4,5]. However, it was revealed that a different type of TPPP also occurs in fungi, the so-called fungal type TPPP, in which the characteristic C-terminal region is present twice [7] (Figure 1). (The figure also shows the location of some characteristic sequences.) Both types are present simultaneously in several fungal species. This motivated the present research to see what the distribution of TPPPs (long, fungal type; maybe others) is like in Opisthokonta. Opisthokonta contains two sister clades: Holomycota (including fungi) and Holozoa (including animals); the latter one contains, in addition to Metazoa (animals), the classes Choanoflagellata, Filisterea, and Ichtyosporea, as well as several species considered *incertae sedis* Holozoa [8]. Understanding the evolution of early-branching Opisthokonts is also important for revealing the genome of the single-celled ancestor of animals, and we will also get closer to the origin of Metazoa. The aim was also to see how specific the occurrence of these proteins is to Opisthokonta. Previous studies suggested that this may be the case, as short TPPP has not been found in Opisthokonta, but only in Chlorophytes, Alveolates, Stramenopiles, and Euglenozoa. Over time, it has also been revealed that there is a very strong correlation between the occurrence of TPPPs and the presence of eukaryotic flagella/cilia [9]. This theory was later extended to the idea that a relationship exists between the occurrence of the p25alpha domain and the presence of a flagellum/cilium [4,7,10,11].

## 2. Materials and Methods

Accession numbers of protein and nucleotide sequences refer to the National Center for Biotechnology Information (NCBI) GenBank database unless otherwise stated. NCBI BLAST was used to search databases (http://www.ncbi.nlm.nih.gov/BLAST/) [12]. Whole sequences of various TPPPs were used as queries against protein and TSA (transcriptome shotgun assemblies) databases to find similar sequences in Opisthokonta using the BLASTP and TBLASTN programs, respectively. In some cases, the EST (Expressed Sequenced Tag) and SRA (Sequence Read Archive) databases were also searched. The queries were Opisthokonta TPPPs belonging to various phyla and classes: *Caenorhabditis elegans* NP_491219 (Nematoda), *Nematostella vectensis* XP_001628751 (Cnidaria), *Amphimedon queenslandica* XP_003384590 (Porifera), *M. brevicollis* XP_001743131 (Choanoflagellata), *Batrachochytrium dendrobatidis* XP_006680205, and *Spizellomyces punctatus* XP_016604112 (Chytridiomycota). If a TPPP was found in a species of a given phylogenetic branch, then its sequence was used as a query within the whole branch. The hits were considered as TPPPs when the BLAST E-score was lower than 1 × 10^−5^ and the query and hit were reciprocal best hits [13,14].

Multiple alignments of sequences were conducted by the Clustal Omega program [15]. Phylogenetic trees were constructed based on the Bayesian and maximum-likelihood (ML) methods. The N-terminal amino acids before the p25alpha domain were trimmed after alignment. Bayesian analysis, using MrBayes v3.1.2 [16], was performed to construct phylogenetic trees. The WAG model [17] with rate variation among sites, allowing for some sites to be evolutionary invariable (WAG+G+I), and default priors were used. Two independent analyses were run with three heated and one cold chain (temperature parameter 0.2) for generations indicated in the figure legends, with a sampling frequency of 0.01, and the first 25% of generations were discarded as burn-in. The two runs were convergent, shown by the fact that the value of PSRF (potential scale reduction factor) [18] approached 1. The MEGA11 program [19] was used for ML analysis. Bootstrap values were calculated by bootstrap analyses of 1000 replicates with the WAG+G+I model.

## 3. Results

### 3.1. Identification of New TPPPs by BLAST Search

#### 3.1.1. TPPPs in Animals and Fungi

The BLAST search for TPPPs in Opisthokonta yielded the following results: The presence of the long TPPP is characteristic of animals. It is present in the main groups of the Bilateria clade, Deuterostomia, Protostomia, Xenacoelomorpha; in the latter (in *Symsagittifera roscoffensis*) it was only found last year. However, there was an unexpected finding. In an annelid (*Lamellibrachia satsuma*, phylum Annelida), a previously unknown type of TPPPs (KAI0217909) was found, in which the entire p25alpha domain is present twice, not just the C-terminal part (‘double long’ TPPP) (Figure S1). This species also contains a ‘traditional’ long TPPP (KAI0228059).

TPPP is also present in early-branching Metazoa (Cnidaria, Ctenophora, Porifera); however, it was still not found in Placozoa.

The Ctenophora data were very new; a TPPP was identified in *Bolinopsis microptera* last year and in *Mnemiopsis leidyi* this year. It has been found as TSA in some other Ctenophora species.

The presence of long TPPP in sponges (Porifera) was previously known but only in the class Demospongiae; more recently (2024), it has also been found in the class Homoscleromorpha (XP_065828532, XP_062500770). However, in addition to these, fungal type TPPP (i.e., the C-terminal part is present twice) has been found in several species of Demospongiae; these proteins were found in 2023–2025. Moreover, a ‘double long’ TPPP (see above) is also present in *Halichondria panicea* (XP_064398936.1) (Figure S1).

The situation is similar with the cnidarians (Cnidaria); here too, the presence of long TPPP was known, but only in the class Anthozoa; last year, it was also found in the classes Hydrozoa (*Hydra vulgaris*) and Scyphozoa (*Rhopilema esculentum*). In the species *Alveopora japonica* (class Anthozoa), a fungal type TPPP (as TSA) has been identified.

We will not discuss fungi in detail here; in the last few years (since 2021), we have discussed the occurrence of long and fungal type TPPPs in detail in several articles [7,10,11,20]. There is no significant new data compared to those articles, except that a double long TPPP (KAJ3407993) has been found in a Chytridiales species and a fungal type TPPPP in the phylum Monoblepharidomycota (*Gonapodya* sp. JEL0774, KAJ3339789); see more details in the Discussion Section.

#### 3.1.2. TPPPs in Deep-Branching Opisthokonts

Among the deep-branching opisthokonts, there are several ones that have lost their flagella: *Capsaspora owczarzaki* (class Filasterea), Ichthyophonida (class Ichthyosporea), Cristidiscoidea, and *Corallochytrium*. We did not find TPPPs, or more broadly, proteins/TSAs containing the p25alpha domain, in these phylogenetic units. The situation is different with flagellated species. Among these, no sequence data are available for the genus *Syssomonas*. Aphelidea used to be considered an early-branching independent Opisthokonta class; however, recently, it was classified as a phylum of Fungi (Aphelidiomycota) [21]; they contain both long and fungal type TPPPs [11].

**Figure 2:**
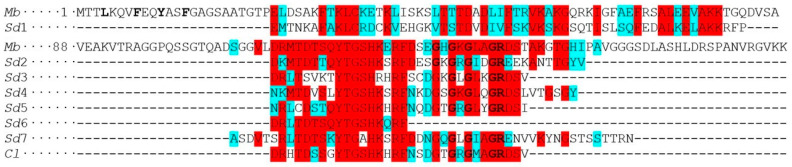
Sequence alignment of *Monosiga brevicollis* (*Mb*) Monbr1/23057 with TPPP-like sequences found in *Sphaerothecum destruens* (*Sd*) SRX737879 and *Corallochytrium limacisporum* (*Cl*) SRX2510794 and SRX2510798. Red and blue background indicate identical and biochemically similar amino acids. The starting sequence of the p25alpha domain (LxxxF(Y)xxFxxF) and the characteristic sequences of the long p25alpha domain (GxGxGxxGR) are indicated in bold.

There are also flagellated species in the classes Ichthyosporea and Filasterea. There was a high probability that TPPPs would be found in these species as well. Although the search in the protein and TSA databases was unsuccessful, the assumption proved to be correct. The complete genome sequences of the flagellated *Ministeria vibrans*, *Pigoraptor vietnamica*, and *Pigoraptor chileana*, belonging to the class Filasterea, have been published [22]. *M. vibrans* possesses a protein that contains the full p25alpha domain, Mvib_g11715, both *Pigoraptor* species have an apicortin (Figure S2). The apicortin is characterized by the presence of two domains: the C-terminal part (30–32 aa) of the p25alpha domain which contains the GXGXGXXGR motif (‘partial p25alpha domain’) and the doublecortin (DCX) domain [5] (Figure S2). *Sphaerothecum destruens* belonging to the Dermocystida order of Ichthyosporea has zoospore with posterior flagellum. Blast search (TBLASTN) of the NCBI SRA database resulted in some partial sequences characteristic for long p25alpha domain containing TPPPs (Figure 2).

Since comparative analyses of the flagellar toolkit showed a previously unnoticed flagellar apparatus in *Corallochytrium limacisporum* [23], thus TBLASTN search was carried out against the NCBI SRA database using various TPPPs and apicortins as queries. There were only hits when long TPPP or apicortin was used as the query. However, we only found a short sequence (32 aa) corresponding to the C-terminus of the p25alpha domain, which is common in the two proteins (Figure 2).

In the class Choanoflagellata, it was known that *M. brevicollis* and *Salpingoeca rosetta* (both species belonging to Craspedida order and Salpingoecidae family) contain long TPPP. In this order/family, we have now found additional long TPPPs, such as TSAs (*Choanoeca perplexa*, *Mylnosiga fluctuans*, *Salpingoeca urceolata*). In addition, some *Salpingoeca* species (*S. kvevrii*, *S. punica, S. dolichothecata*) possess TPPP (as TSA) in which the entire p25alpha domain is present twice (Figure S1). TPPPs have been found in another order of Choanoflagellates, the Acanthoecida, in both families (Acanthoecidae, Stephanoecidae), but they were unexpectedly of a different type: the entire short p25alpha domain occurs twice in them (Figure S1). This overturned the view that opisthokonts do not contain short TPPPs. The same type (‘double short’ TPPP) is found in the Opisthokonta of uncertain phylogenomic classification, in *Tunicaraptor unikontum* (Figure S1).

Then a BLAST search was performed where the queries were the Choanoflagellata TPPPs containing two short p25alpha domains. The best matches were given by two Ciliophora proteins, *Stylonychia lemnae* CDW85805 and *Halteria grandinella* TNV76936, respectively; (query cover 94–97%, percent identity 46–47% and 39–42%, E-value less than 1 × 10^−80^ and less than 1 × 10^−60^). Additional hits were mainly proteins from other Alveolata species. In the case of *Tunicaraptor* TPPP, most hits belonged to the Stramenopiles (Heterokonta), but TPPPs of Chlorophyta and Alveolata species were also abundant among them.

Finally, we investigated whether long or fungal type TPPP occurs outside of Opisthokonta. We found only two sequences; both hits were fungal type TPPPs. One of them is in an Amoebozoa (*Physarum polycephalum* GDRG01010249), the other one is in a Heterolobosea (*Acrasis kona* KAL0476604).

### 3.2. Phylogenetic Analysis

**Figure 3:**
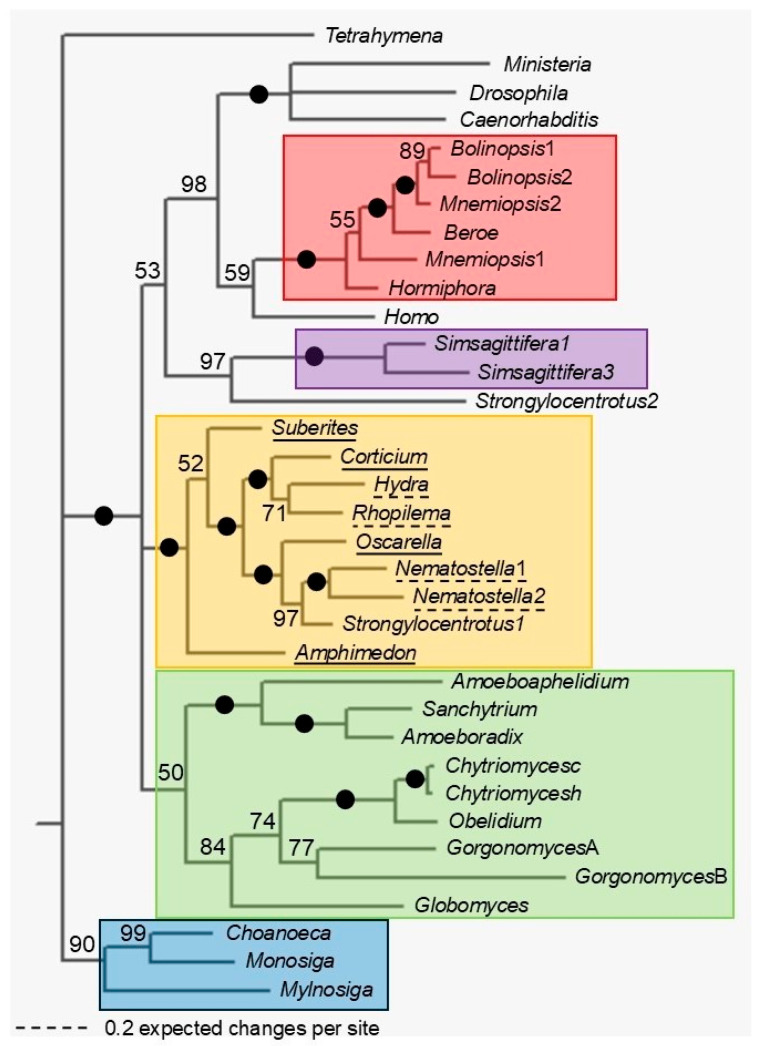
Phylogenetic tree of long TPPPs by Bayesian analysis. Two independent analyses were run for 1.2 × 10^−6^ generations. *Tetrahymena thermophila* short TPPP was used as the out-group. The accession numbers of proteins/TSAs are listed in Table S1. Black circles at a node indicate that the branch was supported by maximal Bayesian posterior probability (BPP). All the other branches were supported by BPP as indicated at the node. Color code: Ctenophora (red), Xenacoelomorpha (lilac), Porifera and Cnidaria (orange), Fungi (green), Choanoflagellata (blue).

**Figure 4:**
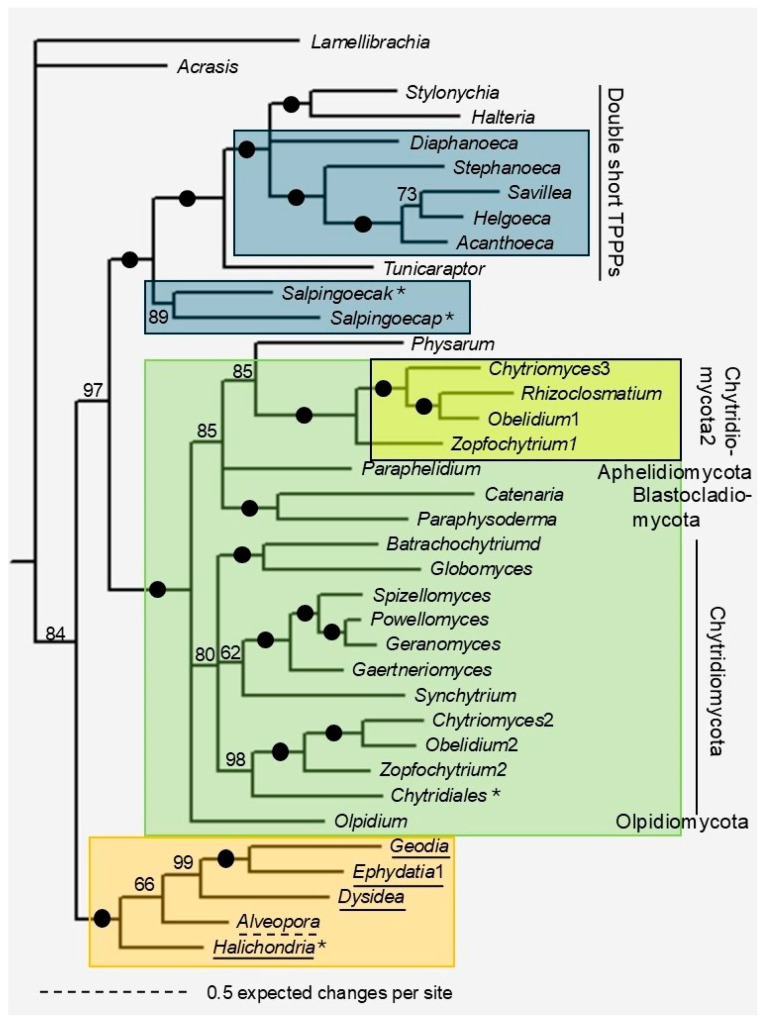
Phylogenetic tree of fungal and ‘double’ type TPPPs by Bayesian analysis. Two independent analyses were run for 2.1 × 10^−6^ generations. The root of the tree was chosen arbitrarily. The accession numbers of proteins/TSAs are listed in Table S2. Black circles at a node indicate that the branch was supported by maximal Bayesian posterior probability (BPP). All the other branches were supported by BPP as indicated at the node. Color code: Porifera and Cnidaria (orange), Fungi (green), Choanoflagellata (blue). A special fungal group (Chytriomycota2) is indicated by a yellow box. Asterisks label double long TPPPs.

Phylogenetic trees were constructed using Bayesian (Figure 3, Figure 4, Figures S4 and S7) and ML (Figures S5 and S8) methods. Trees of long TPPPs can be seen in Figure 3 and Figure S5. Only the p25alpha domains were used in the alignment on which the analyses were based, i.e., the N-termini of various lengths of the proteins were deleted (Figure S3). TPPPs of Fungi, Choanoflagellata, Filasterea, Ctenophora, Porifera, Cnidaria, and Xenacoelomorpha, including the newly identified ones, and a few proteins of Protostomia and Deuterostomia were included in the analysis.

The phylogenetic trees are quite similar; TPPPs of Fungi, Choanoflagellata, Ctenophora, and Xenacoelomorpha form monophyletic clades; however, TPPPs of Porifera and Cnidaria form a common clade, with high support (Bayesian posterior probability, BPP = 1, bootstrap value, BS = 96%). The other clades generally also have high support, except Fungi (BPP = 0.5, BS = no), with Choanoflagellata: BPP = 0.9, BS = 90%; Ctenophora: BPP = 1, BS = 100%; Xenacoelomorpha: BPP = 1, BS = 100%). 

The position of the Filasterea *M. vibrans* protein is surprising. In a few cases two TPPPs from the same species were included in the analysis. According to the trees, these pairs are generally the results of intraspecies duplication; however, in other cases they are more distantly related, e.g., *Strongylocentrotus purpuratus* paralogs are in different clades.

Trees of the fungal and ‘double’ type TPPPs can be seen in Figure 4 and Figure S8. The N-termini of various lengths of the proteins were deleted in the alignment (Figure S6). The clades are arranged according to the type of TPPP and the taxonomy. The double short TPPPs form a well-supported clade (BPP = 1, BS = 92%). The Porifera and Cnidaria TPPPs form one clade here too, like in the long TPPP tree (support: BPP = 1, BS = 67%). The TPPPs of Fungi (all of them are fungal type ones) form a clade with high and moderate support (BPP = 1, BS = 51%). Within the clade, the phyla are distinguished as Aphelidiomycota, Blastocladymycota, Olpidiomycota, and Chytridiomycota; within the latter, the classes (Spizellomycetes, Chytridiomycetes, Rhizophydiomycetes, and Synchytriomycetes) are also separated. In line with previous publications [7,11], within Fungi, a clade (Chytridiomycota2) containing paralogs of Chytridiomycetes has been revealed, with maximal support on both trees. The *Physarum* fungal type TPPP is a sister to this group in both trees.

## 4. Discussion

The new data further strengthen the observation that flagellated species possess (a) p25alpha domain-containing gene/protein(s). No such gene/protein has been found in opisthokonts that lost their flagella (some species of Filasterea and Ichthyosporea, Cristidiscoidea, and terrestrial fungi), while in those with flagella, provided that sequence data are available, it was found in all of them. Of the so far known TPPP types characteristic of opisthokonts, the ‘long type’ is the most common, absent only in placozoans and *Tunicaraptor* (Table 1); instead, these two species have a different p25alpha domain-containing protein that is likely capable of performing the same microtubule stabilizing function as the long TPPP. The ‘fungal type’ TPPP was previously known only in flagellated (non-terrestrial, early-branching) fungi; the present study has revealed that it can also be found in sponges and cnidarians. It occurs together with the long TPPP in both aforementioned animal phyla and in fungi; this finding is generally, but not always, true even when broken down by species. Five fungal phyla are known to harbor fungal type TPPP: Aphelidiomycota, Monoblepharidomycota, Chytridiomycota, Blastocladiomycota, and Olpidiomycota [7,10,11] (Figure 4). Some Chytridiomycota species also possess two such paralogs that share approximately 40–45% of their identity [7].

Fungal type TPPPs contain the highly conserved C-terminal part of the ‘long’ p25alpha domain twice. We have now also found TPPPs in which the entire p25alpha domain is present twice (Figure S1). This is much rarer; it was found in four distant phyla, each in only one genus. The three cases are quite different. The Annelida *L. satsuma* also has a conventional long TPPP (KAI0228059), which shares about 40% identity with both halves of the ‘double long’ TPPP. The Porifera *H. panicea* XP_064398936 appears to be the only TPPP in the species. Some species of the Choanoflagellata genus, *Salpingoeca*, have only long TPPPs, while other species have only a double long type one. *Chytridiales* sp. JEL 0842 possesses a fungal type TPPP, too. Based on the phylogenetic trees, it appears that in all cases the emergence of this TPPP type is the result of independent, in-species or in-genus gene duplication.

Choanoflagellates possess the most types of proteins containing the p25alpha domain (Table 1). The occurrence of these proteins seems to be order- and family-dependent. The long and double long TPPPs discussed so far occur in the order Craspedida and the family Salpingoecidae (this is the only extant family of the given order). Fungal type TPPP, based on the data so far, is not found in Choanoflagellata. In the other order of Choanoflagellata, Acanthoecida, none of the three types of TPPPs mentioned have been found. Instead, there is a type previously unknown in Opisthokonta, the ‘double short’ TPPP. It is found in both families of this order (Acanthoecidae and Stephanoecidae). It is very interesting that apicortin is also found in the latter family [24]. As already mentioned, apicortin contains only the C-terminal part of the p25alpha domain and has also a DCX domain [5]. What is particularly interesting is that the short and double short TPPPs differ from the long and double long TPPPs in that they lack the C-terminal part of the p25alpha domain.

In the case of other smaller Opisthokont groups, our knowledge is somewhat limited, but more and more new, complete or partial genomes are becoming known [22,23,25,26]. These data seem to confirm that the presence of the p25alpha domain is linked to the eukaryotic flagellum/cilium. No protein/nucleotide containing such a domain has been found in non-flagellated Ichthyosporea and Filasterea species. However, the flagellated Filasterea species *P. vietnamica*, *P. chileana*, and *M. vibrans* possess such a protein. *Pigoraptor* species contain apicortin, while *M. vibrans* contains a protein that includes a partial PHA03247 domain in addition to the full-length p25alpha domain (Figure S2). As shown in Figure 3, this protein (the analysis only covered the p25alpha domain, not the entire protein) is found in a surprising place in the long TPPP family tree, in a clade of animal (Bilaterian and Protostomian) species. Before considering horizontal gene transfer (HGT), we should consider that the BLAST search showed that the homologues with the most similar sequence to the p25alpha domain of the protein were found in birds, namely in the order Galliformes. (*Ministeria* lives in marine water [27].)

BLAST searches of NCBI SRA databases suggest that the flagellated Ichthyosporea *S. destruens* also likely contains a long or fungal type or double long TPPP, but the complete sequence is not yet known (Figure 2). The situation is even more uncertain in the case of the Opisthokonta *incertae sedis Corallochytrium*, where SRA database searches identified only a short (32aa) sequence, which is homologous to both long TPPP and apicortin sequences (Figure 2). Although the presence of a flagellum in this species is morphologically ambiguous, comparative genomic analysis revealed most of the genes of the flagellar toolkit [23].

Double short TPPP is found in an Opisthokonta of uncertain phylogenomic classification, *T. unicontum*, discovered a few years ago [25]. This TPPP shares 35–40% identity and 46–53% similarity with Choanoflagellata TPPPs. The fact that both Choanoflagellata and *Tunicoraptor* contain this gene/protein suggests that it may have been present in the common ancestor of Holozoa (defined in [28]), which includes animals, choanoflagellates and several other clades (Filasterea, Ichthyosporea, *Corallochytrium*, *Syssomonas*, and *Tunicaraptor*) [8,25]. Although double short TPPPs have not been studied in detail, their occurrence is similar to that of short TPPPs [4]. A quick BLAST search showed that they are mainly found in Stramenopiles (Heterokonta), Alveolates (Ciliophora and Myzozoa), and Chlorophyta, but also in Discoba (Euglenozoa). This means that they occur in the main branches of the multi-supergroup ‘Diphoda+’ [29,30] and, until now, they have been thought to be absent in the other one, Opimoda+, which Opisthokonta belongs to. The BLAST search revealed that among ‘Opimoda+’, only Choanoflagellates and *Tunicaraptor* have this type of TPPP. What does this mean? Since double-short TPPP is present in several related species, possible genome contamination or independent HGTs can be ruled out. This gene should be present in the last common ancestor of Holozoa. Several groups of Holozoans have lost their flagella, and therefore their TPPP. Of those that have retained them, Metazoa (animals) and Fungi lack this protein, due to gene loss. It may provide important information if data from the *Syssomonas* (also flagellated) genome becomes available. How did the Holozoa ancestor acquire double short TPPP? There are two possibilities. One of them is an ancient HGT. The other one is that it was present in the last eukaryotic common ancestor and was retained in Diphoda+ clades but was lost in Opimoda+ clades except Holozoa. This would mean several independent gene losses among Opimoda+.

The BLAST search yielded another unexpected result. The presence of the long p25alpha domain has so far been associated with Opisthokonta. This domain is present in the long, fungal, and newly identified double long types of TPPPs. It is still true that the long and double long TPPPs have not been found outside Opisthokonta, but this statement seems to be refuted by the occurrence of the fungal type TPPP, and two new, non-opisthokont TPPPs were identified. Less surprising is its occurrence in the Amoebozoa *P. polycephalum*. It is an amoeba that can differentiate reversibly into a flagellated form. It was suggested that Amoebozoa, together with Obazoa, which also contains Opisthokonta, forms a taxonomic supergroup, Amorphea [31]. This view was later refined, and it was suggested that both Amoebozoa and Opisthokonta belong to Opimoda+, one of the two multi-supergroups [29,30]. This suggests that the long p25alpha domain was present in the last common ancestor of Opimoda+.

In the phylogenetic trees, *P. polycephalum* is sister (high support in the Bayesian tree, lower support in the ML tree) to a well-defined Fungi clade, which is close to the root of the fungal clade—only the recently classified Aphelidiomycota [21] is closer—and is sister to all other fungi. The existence of this clade has been previously observed [7,11], and its members belong to the class Chytridiomycetes; within it is the order Chytridiales, and their sequences differ significantly from those of other fungal TPPPs. In the species in which they occur, the other, ‘traditional’ fungal type TPPP paralog is also present. Until now, it was likely that there was a duplication event within the class or order. Now, the possibility arises that these paralogs are some kind of relict. Alternatively, HGT could be another option. Early diverging fungi are characterized by higher HGT rates compared to terrestrial fungi; Neocallimastigomycota and Chytridiomycota acquired genes most frequently in this way [32]. Species of Chytridiales were shown to receive genes from Amoebozoa as well [32]; this fact may support HGT in the given case, too.

The presence of a fungal type TPPP in the Heterolobosea *A. kona* (KAL0476604) fits much less well into the picture because of two reasons. First, although the life cycles of many Heterolobosea alternate between flagellate and amoeboid stages, *A. kona* has been considered as an aflagellate species. However, very recently, genetic evidence for cryptic flagellate stages in presumably aflagellate Heterolobosea has been found, including in *A. kona* and *A. rosea* [33]. Genes of flagellum-associated proteins, e.g., responsible for intraflagellar transport, were identified in these species [34].

**Figure 5:**
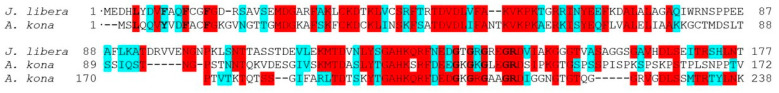
Sequence alignment of *J. libera* (EC692700) and *A. kona* (KAL0476604) TPPPs. Red and blue backgrounds indicate identical and biochemically similar amino acids.

Second, Heterolobesea belongs to Diphoda+. Species in this multi-supergroup have been known to contain no long p25alpha domain. Heterolobosea is part of a larger phylogenomic clade, named Discoba, formed by Jakobids, Euglenozoa, and Heterolobosea, suggested in 2009 [35]; later, other groups were included as well. No other TPPPs (protein/TSA) were found in Heterolobosea; this also holds true for Jakobida. Euglenozoa have long been known to have genera containing short TPPPs (*Trypanosoma*, *Leishmania*) [4]; now a double short protein has also been found (*Diplonema papillatum* KAJ9468261). This fits with the idea that short/double short TPPPs are characteristic of Diphoda+. However, when the BLAST search was extended to ESTs, we found that among the Jakobida *Jakoba libera* sequences, there was one that contained a complete p25alpha domain. The exact type of TPPP this sequence corresponds to (long, fungal, double long) is not clear, since there is no stop codon in the nucleotide sequence. Based on the known sequence, it may be a fungal type. When this sequence was used as a query in a protein BLAST search, it gave interesting results. The first 100 hits, aside from 2, were all Opisthokonta (animal and fungi) long and fungal type TPPPs. One exception was the above-mentioned *A. kona* fungal type TPPP, which showed the highest sequence identity (44.74%) to the translated sequence of the *J. libera* EST (EC692700) (Figure 5). This supports the idea that the *A. kona* TPPP was a real hit and that the two Discoba hits were mutually reinforcing. It is conceivable that the last eukaryotic common ancestor contained a TPPP that had a complete p25alpha domain. This was retained in Diphoda+ close to the root, while it was lost in other clades, many of them losing also a flagellum.

The other exception was a big surprise: *Cymbomonas tetramitiformis* KAK3252176. *C. tetramitiformis* is a green alga (phylum Clorophyta) that is also a member of Diphoda+ but far from the root. Moreover, the hypothetical protein contains three full p25alpha domain so it can be considered as a ‘triple long’ TPPP. This kind of TPPP has not been known at all. Clorophyta species are mostly flagellated, and many of them possess TPPPs or TPPP-like proteins; however, these proteins contain either a short p25alpha domain (without the C-terminal part) or partial domains (only the C-terminus).

## 5. Conclusions

**Figure 6:**
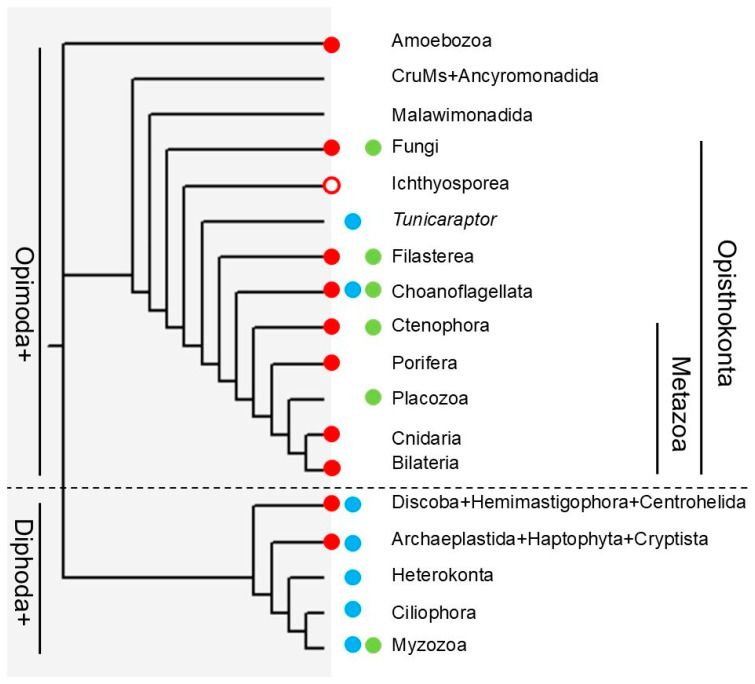
Occurrence of TPPPs/p25alpha domains. The phylogenetic tree is based on Ref. [30]. Red, blue and green circles indicate the presence of TPPPs with whole p25alpha domain (long, fungal, and double long TPPPs), TPPPs with p25alpha domain missing the C-terminus (short and double short TPPPs), and apicortin (with C-terminal of p25alpha domain plus DCX domain), respectively. An open red circle indicates that a long TPPP is probably present. Some lineages may not have sufficient data to definitively show absences. The exact position of *Tunicaraptor* is uncertain.

First, new types of TPPPs were found in which the whole p25alpha domain was present in duplicate or triplicate (double long TPPP and triple long TPPP). Additionally, the results suggest that we should change our view concerning the phylogenomic distribution of TPPPs (Figure 6).

In general, the previous view was that TPPPs containing the whole p25alpha domain are present in Opimoda+; TPPPs containing a shorter p25alpha domain missing the C-terminus can be found in Diphoda+. Now, we have identified a TPPP in choanoflagellates and in the uncertainly classified Opisthokonta *Tunicaraptor* that contains the short p25alpha domain in duplicate, which was previously known only in the Diphoda+ clade. On the other hand, we found an Opisthokonta (Opimoda+)-specific ‘fungal type’ TPPP in a Heterolobosea (Diphoda). Moreover, fungal type TPPPs, which contain a whole p25alpha domain and additionally its C-terminal part, were not found in fungi only, as has been previously thought, but also in animals and Amoebozoa and the afore-mentioned Heterolobosea. Based on these results, we need to rethink the evolutionary history of TPPPs. It seems very probable that the last eukaryotic common ancestor possessed proteins containing a whole p25alpha domain and a short p25alpha domain, respectively.

## Figures and Tables

**Table 1:** Occurrence of p25alpha domain-containing proteins in Opisthokonta.

Phylogenetic Unit	TPPP				Apicortin
	Long	Fungal Type	Double Long	Double Short	
Bilateria	Yes		Yes		
Cnidaria	Yes	Yes			
Ctenophora	Yes				Yes
Placozoa					Yes
Porifera	Yes	Yes	Yes		
Fungi	Yes	Yes			Yes
Choanoflagellata	Yes		Yes	Yes	Yes
Filasterea	Yes				Yes
*Tunicaraptor*				Yes	

## Data Availability

The data presented in this study are available in this paper and in the Supplementary Material.

## Supplementary Materials

The following supporting information can be downloaded at https://www.mdpi.com/article/10.3390/biology14121784/s1: Table S1: Accession numbers of proteins/TSAs/WGSs shown in Figure 3 and Figure S6. Table S2: Accession numbers of proteins/TSAs/WGSs shown in Figure 4 and Figure S8. Figure S1: Duplication (triplication) of p25alpha domain in ‘double (triple) type’ TPPPs. Figure S2: Newly identified p25alpha domain containing proteins in class Filasterea. Figure S3: Multiple alignment of long type TPPPs. Figure S4: Bayesian tree for Figure 3. Figure S5: ML tree of long TPPPs. Figure S6: Multiple alignment of fungal and ‘double’ type TPPPs. Figure S7: Bayesian tree for Figure 4. Figure S8: ML tree of fungal and ‘double’ type TPPPs.

## Abbreviations

The following abbreviations are used in this manuscript:

BLAST	Basic Local Alignment Search Tool
EST	Expressed Sequenced Tag
DCX	Doublecortin
HGT	Horizontal Gene Transfer
ML	Maximum Likelihood
NCBI	National Center for Biotechnology Information
SRA	Sequence Read Archive
TSA	Transcriptome Shotgun Assembly
TPPP	Tubulin Polymerization Promoting Protein
WGS	Whole Genome Shotgun
