# Supplementary material for: Rethinking the Evolution of Tubulin Polymerization Promoting Proteins
Source: Biology (Basel). 2025 Dec 14;14(12):1784. doi: 10.3390/biology14121784 (PMC12731214; doi:10.3390/biology14121784)
Supplement: Supplementary file 1 [file biology-14-01784-s001.zip › biology-4001518-supplementary.pdf]

Table S1. **Accession Numbers of proteins/TSAs/WGSs shown in Figures 3 and S5**

Name on Figures 4, 5	Species	Phylum (*Class)	NCBI Accession Number		
			Protein	TSA	WGS
Tetrahymena	*Tetrahymena thermophila*	Ciliophora	XP_001023601		
Choanoeca	*Choanoeca perplexa*	Choanoflagellata*		GGOP01010367	
Mylnosiga	*Mylnosiga fluctuans*	Choanoflagellata*		GGOI01006322	
Monosiga	*Monosiga brevicollis*	Choanoflagellata*	Monbr1/23057[1]		
Ministeria	*Ministeria vibrans*	Filisterea*	Mvib_g11715[2]		
Amoeboaphelidium	*Amoeboaphelidium protococcarum*	Aphelidomycota	KAI3650757		
Chytriomycesc	*Chytriomyces confervae*	Chytridiomycota	TPX78276		
Chytriomycesh	*Chytriomyces hyalinus*	Chytridiomycota	KAI8827812		
Obelidium	*Obelidium mucronatum*	Chytridiomycota	KAI9342551		
Globomyces	*Globomyces pollinis-pini*	Chytridiomycota	KAI8895260		
GorgonomycesA	*Gorgonomyces haynaldii*	Chytridiomycota	KAI8912823		
GorgonomycesB	*Gorgonomyces haynaldii*	Chytridiomycota	KAI8906053		
Sanchytrium	*Sanchytrium tribonematis*	Sanchytriomycota			JADGIG010000129
Amoeboradix	*Amoeboradix gromovi*	Sanchytriomycota			JADGIF010000946
Bolinopsis1	*Bolinopsis microptera*	Ctenophora	XP_063688086		
Bolinopsis2	*Bolinopsis microptera*	Ctenophora	XP_063688098		
Beroe	*Beroe forskalii*	Ctenophora		GHXY01036189	
Mnemiopsis1	*Mnemiopsis leidyi*	Ctenophora	KAL5261959		
Mnemiopsis2	*Mnemiopsis leidyi*	Ctenophora	KAL5261887		
Hormiphora	*Hormiphora californensis*	Ctenophora		GGLO01026289	
Suberites	*Suberites domuncula*	Porifera	ADX30619		
Amphimedon	*Amphimedon queenslandica*	Porifera	XP_003384590		
Corticium	*Corticium candelabrum*	Porifera	XP_062500770		
Oscarella	*Oscarella loburalis*	Porifera	XP_065828532		
Nematostella1	*Nematostella vectensis*	Cnidaria	XP_001628751		
Nematostella2	*Nematostella vectensis*	Cnidaria	XP_001641058		
Hydra	*Hydra vulgaris*	Cnidaria	XP_047138925		
Rhopilema	*Rhopilema esculentum*	Cnidaria	XP_065065091		
Symsagittifera1	*Symsagittifera roscoffensis*	Xenacoelomorpha	XP_063715394		
Symsagittifera3	*Symsagittifera roscoffensis*	Xenacoelomorpha	XP_063727536		
Drosophila	*Drosophila melanogaster*	Arthropoda	NP_648881		
Caenorhabditis	*Caenorhabditis elegans*	Nematoda	NP_491219		
Strongylocentrotus1	*Strongylocentrotus purpuratus*	Echinodermata	XP_782492		
Strongylocentrotus2	*Strongylocentrotus purpuratus*	Echinodermata	XP_793987		
Homo	*Homo sapiens*	Chordata	NP_057048		

[1]http://genome.jgi-psf.org/Monbr1/Monbr1.home.html. [2]Ref. [22].

Table S2. Accession Numbers of proteins/TSAs/WGSs shown in Figures 4 and S8

Name on Figures 4, 5	Species	Phylum (*Class)	NCBI Accession Number		
			Protein	TSA	WGS
Acrasis	*Acrasis kona*	Heterolobosea	KAL0476604		
Physarum	*Physarum polycephalum*	Evosea (Amoebozoa)		GDRG01010249	
Stylonychia	*Stylonychia*	Ciliophora			
Halteria	*Halteria*	Ciliophora			
Tunicaraptor	*Tunicaraptor unikontum*			GIQG01040147	
Salpingoecak	*Salpingoeca kvevrii*	Choanoflagellata*		GGOX01011241	
Salpingoecap	*Salpingoeca punica*	Choanoflagellata*		GGOZ01013784	
Diaphanoeca	*Diaphanoeca grandis*	Choanoflagellata*		GGPB01024076	
Stephanoeca	*Stephanoeca diplocostata*	Choanoflagellata*		GGOM01054003	
Savillea	*Savillea parva*	Choanoflagellata*		GGOL01030293	

Helgoeca	*Helgoeca nana*	Choanoflagellata*		GGOR01010129	
Acanthoeca	*Acanthoeca spectabilis*	Choanoflagellata*		GGPA01008943	
Paraphelidium	*Paraphelidium tribonematis*	Aphelidomycota	TRINITY_DN24782[1]		
Spizellomyces	*Spizellomyces punctatus*	Chytridiomycota	XP_016604112		
Powellomyces	*Powellomyces hirtus*	Chytridiomycota	KAI8908161		
Chytriomyces2	*Chytriomyces confervae*	Chytridiomycota	TPX65886		
Chytriomyces3	*Chytriomyces confervae*	Chytridiomycota	TPX72533		
Chytridiales	*Chytridiales* sp, JEL 0842	Chytridiomycota	KAJ3407993		
Rhizoclosmatium	*Rhizoclosmatium globosum*	Chytridiomycota	ORY45507[2]		
Synchytrium	*Synchytrium microbalum*	Chytridiomycota	XP_031024160		
Geranomyces	*Geranomyces variabilis*	Chytridiomycota	KAJ3155973		
Gaertneriomyces	*Gaertneriomyces semiglobifer*	Chytridiomycota	Gaesem1531638[2]		
Obelidium	*Obelidium mucronatum*	Chytridiomycota	KAI9342285	Obemuc1859513	
Obelidium	*Obelidium mucronatum*	Chytridiomycota	KAI9351240	Obemuc1832726	
Globomyces	*Globomyces pollinis-pini*	Chytridiomycota	Glopol1609812[2]		
Zopfochytrium1	*Zopfochytrium polystomum*	Chytridiomycota	KAI9325442		
Zopfochytrium2	*Zopfochytrium polystomum*	Chytridiomycota	KAI9357369		
Batrachochytriumd	*Batrachochytrium dendrobatidis*	Chytridiomycota	XP_006680205		
Catenaria	*Catenaria anguillulae*	Blastocladiomycota	ORZ35986		
Paraphysoderma	*Paraphysoderma sedebokerense*	Blastocladiomycota	KAI9140125		
Olpidium	*Olpidium bornovanus*	Olpidiomycota	KAG5460860 +<br>KAG5458366[3]		JAEFCI010004592+<br>JAEFCI010008581
Geodia	*Geodia barretti*	Porifera	CAI8012306		
Dysidea	*Dysidea avara*	Porifera	XP_065913653		
Halichondria	*Halichondria panicea*	Porifera	XP_064398936		
Ephydatia1	*Ephydatia muelleri*	Porifera	KAL5467498		
Alveopora	*Alveopora japonica*	Cnidaria		GGJR01215658	
Lamellibrachia	*Lamellibrachia satsuma*	Annelida	KAI0217909		

[1]Torruella, G. Commun_Biol_Aphelid_Datasets. Figshare. Dataset. 2018. Available online: doi:10.6084/m9.figshare. 7339469.v1 (accessed on 14 November 2022).

[2]https://mycocosm.jgi.doe. gov/mycocosm/home. [3]Sequence reconstructed in Ref. [10].

Figure S1: Duplication (triplication) of p25alpha domain in 'double (triple) type' TPPPs

The two (three) halves of the sequences were aligned by ClustalOmega [15]. Identical and biochemically similar amino acids are indicated by * and ':', respectively.

The starting sequence of the p25alpha domain (**L**xxx**F**(**Y**)xx**F**xx**F**) and the characteristic sequences of the long (**G**x**G**x**G**xx**GR**) and short (**GGP**) domains are indicated in bold.

Cymbomonas tetramitiformis **KAK3252176** Chlorophyta

>1
1MATMLELFTQFASFGSQSVSTEMDGPKFAKMCRDTRLLDKKFTTTDADIIFAKVKAKGLRRIDYAQFCVALTLIADRKKVSEDSIRQLLLG
DSSGPLVAQATKIEHSGVYDRLTDSAKYTGAHKERFDEEGHGRGLSGRDVGGKGDGHVPAMGTFGTSTDNSPRRTVFGSAASTEESPASTVP
QRNVKFQEPPKPTPTESSAPPLRGNNADPGNSSTVN 219
>2
220LVEVFTAYASFGKGAIVADMDGPRFAKLCCECAMLDKLFTATDADLTFAKVKQKGQRKIGYRQFCEALAIAADKKQVHMEDLMHGMLAG
AQ PLVEQMTPVE NDSEENDLVE
NDSRHRWKTTMKRLMDTTQYTGAHRERFDEEGKGRGALGRELGGKGTGHVPAMGVMGPQYTGSSTAVTPPPRALWRDLISKSQSQASSQSGM
STPCSGATHDEGSLGGLQDASEGQDDEFNLES 454
>3
455IRDVFTTFSSFRGGNATSAPGQEMDGPKFAKLCRDCKLIDKLFTVTDADLTFAKVKVKGQRKIRFEQFCDAVYIMAEKKRVEVPKLVTR
ITKHGVP TVSGAKAETG GLLDKLTDVSLYGGAHKERFDEHGHGRGVQGRDAIGQSPLLLRPTPGHGSTWGHLDEHAPHT 622

```
1       MATMLELFTQFASFGS----QSVSTEMDGPKFAKMCRDTRLLDKKFTTTDADIIFAKVKA
2       ---LVEVFTAYASFGKG----AIVADMDGPRFAKLCCECAMLDKLFTATDADLTFAKVKQ
3       ---IRDVFTTFSSFRGGNATSAPGQEMDGPKFAKLCRDCKLIDKLFTVTDADLTFAKVKV
           : ::** ::**       :   :****:***:* :  ::** **.****: *****
```

```
1       KGLRRIDYAQFCVALTLIADRKKVSEDSIRQLLLGDSSG---------------PLVAQA
2       KGQRKIGYRQFCEALAIAADKKQVHMEDLMHGMLAGAQPLVEQMTPVENDSEENDLVEND
3       KGQRKIRFEQFCDAVYIMAEKKRVEVPKLVTRIT--KHG---------------VPTVSG
        ** *:* : *** *: : *::*:*   .:   :                       . .

1       TKIEHSGVYDRLTDSAKYTGAHKERFDEEGHGRGLSGRDVGGKGDGHVPAMG----TFGT
2       SRHRWKTTMKRLMDTTQYTGAHRERFDEEGKGRGALGRELGGKGTGHVPAMGVMGPQYTG
3       AKAETGGLLDKLTDVSLYGGAHKERFDEHGHGRGVQGRDAIGQSPLLLRPTPGHGSTWGH
        :: .     .:* * : * ***:*****.*:***  **:  *:.   :         :

1       STDN--SPRRTVFGSAASTEESPASTVPQRNVKFQEPPKPTPTESSAPPLRGNNADPGNSSTVN--------------
2       SSTAVTPPPRALWRDLISKSQSQASS----------------QSGMSTPCSGATHDEGSLGGLQDASEGQDDEFNLES
3       LDEH--APHT--------------------------------------------------------------------
               *  ::: .  *..:* **:                 .. : *  * . * *.   ::
```

Lamellibrachia satsuma **KAI0217909.1** Annelida

>1

1MASGGDIKAAFKLFATKNNGAEATSKDITRWCTDAGVFGKTCNSNNLDIAFSKVKPKGKNTITAKEIDALIAELAKKYKDDKKCDEAEAVS
KINEKMSNAVPKGHGTTKTSKTGNVGKMTDTSQYTGSHKERFDADGKGKGIEGRENLADNSGYVGNYKGSG*T*GYPLITSWVKNVPSNPKLLC
RLSSRDIHVTQTS 196

>2

197EMGDLATTFKSFATKANGAEATTKDITRWFTDCGILTKKSCNSNNLDICFSKVKQKGK*N*TITSKEMTALIAEVAKAYKTDHKLGSDGEA
VAQMTEKLAGGKPMAHGTTKASATGGVDRMTDTSKYTGSHKERFDAEGKGKGMAGREDLHDNSGYVGAYK*D*AYSSRRRRFDEAECSRKYTMF
AGRTQLRLVIPVHHSLVGPSDKQRVNTGSSTAKNLVDVDSG 418

```
1       MASGGDIKAAFKLFATKNNGAEATSKDITRWCTDAGVFG-KTCNSNNLDIAFSKVKPKGK
2       --EMGDLATTFKSFATKANGAEATTKDITRWFTDCGILTKKSCNSNNLDICFSKVKQKGK
            **: ::** **** ******:****** **.*::  *:********.***** ***
1       NTITAKEIDALIAELAKKYKDDKKC-DEAEAVSKINEKMSNAVPKGHGTTKTSKTGNVGK
2       NTITSKEMTALIAEVAKAYKTDHKLGSDGEAVAQMTEKLAGGKPMAHGTTKASATGGVDR
        ****:**: *****:** ** *:*  .:.***:::.**::.. * .*****:* **.*.:

1       MTDTSQYTGSHKERFDADGKGKGIEGRENLADNSGYVGNYKGSGTGYPLI----------
2       MTDTSKYTGSHKERFDAEGKGKGMAGREDLHDNSGYVGAYKDAYSSRRRRFDEAECSRKY
        *****:***********:*****: ***:* ******* **.: :.

1       ---TSWV---KNVPSNPKLLCRLSSRDIHVTQTS----------
2       TMFAGRTQLRLVIPVHHSLVGPSDKQRVNTGSSTAKNLVDVDSG
           :. .     :* : .*:   ..: ::. .::
```

Halichondria panicea **XP_064398936.1** Porifera; Demospongiae

>1

1MSLESVFKNFCSFGAGKDGKAQMDNAKFAKLFRDLKLLDKKFTATDVDIIFSKTEVKPKAERKINFEQFRKAVMLAAEIKYPGDADCVKKL
EGKIISGAGPGSSSPSAKVSAASGSVVDRLTDTSKYTGSHKERFDASGKGKGIEGRADPAAGSGNGYVSGYKAGGSYGKVNKHCGEKGKSRS
TTTHWN 189

>2

190MSLESVFKSFCSFGAGKDGKAQMDNAKFAKLFRDLKLLDKKLTATDVDIIFSKTEVKPKAERKINFEQFRKAIKLAAEKKYPGDADCVK
KLEGKIISGEGPGSSSPSARVRTASGGVVDRLTDTTKYTGSHKERFDGTGKGKGLEGRDSSAKGRGMVAGSVADQAAYVSGYKAEGSYGKK
369

```
1       MSLESVFKNFCSFGAGKDGKAQMDNAKFAKLFRDLKLLDKKFTATDVDIIFSKTEVKPKA
2       MSLESVFKSFCSFGAGKDGKAQMDNAKFAKLFRDLKLLDKKLTATDVDIIFSKTEVKPKA
        ********.********************************:******************
1       ERKINFEQFRKAVMLAAEIKYPGDADCVKKLEGKIISGAGPGSSSPSAKVSAASGSVVDR
2       ERKINFEQFRKAIKLAAEKKYPGDADCVKKLEGKIISGEGPGSSSPSARVRTASGGVVDR
        ************: **** ******************* *********:* :***.****

1       LTDTSKYTGSHKERFDASGKGKGIEGRADPAAGSG---------NGYVSGYKAGGSYGKVNKHCGEKGKSRSTTTHWN
2       LTDTTKYTGSHKERFDGTGKGKGLEGRDSSAKGRGMVAGSVADQAAYVSGYKAEGSYGKK------------------
        ****:***********.:*****:*** . * * *          .******* *****
```

Salpingoeca punica **GGOZ01013784.1** Choanoflagellata Craspedida Salpingoecidae

>1

1MEDAFVNFASFGSGGKKLHDITLTNYLKMLKATGIIDKHYPTVDAELLFAKGGSKTMSFDKFRQalatiaaakkttveaLEEALVasggps
asgtsptksaVVDRLTDTSQYTGAHKERFDADGKGKGLAGRESVSKGAGALPNKSLNPGPGHVRDLSELTDRTPSDVRGVKYAEGESPPQRK
STSSTVISSPEAKS 197

>2
198LDELFHRFCSISGGSSNKEMDGSKFAKLCRDCGLIGKKLTATDVDLIFTKCKDKSARKIDMQHFNKAIHLIAEHMHLPFDEVEQMMLHA
GGPKLTGTAADNAHIVDKLTDTHGYTGAHKERFDADGKGKGLEGRVQATGTADLSDLTDRTAADVRGVKKH 357

1 **M**EDA**F**VN**F**AS**F**GSGGKKLHDITLTNYLKMLKATGIIDKHYPTVDAELLFAKGGSKT---M
2 **L**DEL**F**HR**F**CS**I**S-GGSSNKEMDGSKFAKLCRDCGLIGKKLTATDVDLIFTKCKDKSARKI
::: * .*.*:. **.. ::: ::: *: : *:*.*: :.*.:*:*:* .*: :

1 SFDKFRQALATIAAAKKTTVEALEEALVASGGPSASGTSPTKSAVVDRLTDTSQYTGAHK
2 DMQHFNKAIHLIAEHMHLPFDEVEQMMLHAGGPKLTGTAADNAHIVDKLTDTHGYTGAHK
.:::*.:*: ** : .: :*: :: :***. :**: :: :**:**** ******

1 ERFDAD**GKGKGLAGR**ESVSKGAGALPNKSLNPGPGHVRDLSELTDRTPSDVRGVKYAEGESPPQRKSTSSTVISSPEAKS
2 ERFDAD**GKGKGLEGR**VQATG----------------TADLSDLTDRTAADVRGVKKH-----------------------
************ ** ..: . ***:***** :******

Salpingoeca kvevrii **GGOX01011241.1** Choanoflagellata Craspedida Salpingoecidae
>1
1MEELFSRFAAFGAGAAATAGELDGAKFAKLCRDTKIISKKTSSTDVDIIFSKVKPKTARKINYEQFLQALDLIAAQKGMTVERLHEKMLEN
AAPTNSGTASANSSIVDKLTDSSLYTGAHKERFDADGKGRGLAGRDSTAKGAGHVGGVHPGGGGVKDLSELLDRSTADVRGVKHDQHDDGKG
TMRAAAPAAAATTSARTSSASAPSGGSPKKRPTTSGSASSGGPSGGTVVKGSSDGYDS 241
>2
242LEELFQSFASFGVGSSAKVKEIDGAKFVKLCKDCKLVDKKTTTTDVDIIFARCKPKGARKANYEQFVHALELIAEQRKTTVEKLHAKLL
KAEGPRLSGTIADQGGVLDKLTDSSLYTGAHKHRFNEDGKGRGIAGRTANEGVKDMSHVLDRSEADVRGVVPRAE 405

1 **M**EEL**F**SR**F**AA**F**GAGAAATAGELDGAKFAKLCRDTKIISKKTSSTDVDIIFSKVKPKTARK
2 **L**EEL**F**QS**F**AS**F**GVGSSAKVKEIDGAKFVKLCKDCKLVDKKTTTTDVDIIFARCKPKGARK
:****. **:**.*::*.. *:*****.***:* *::.***::*******:: *** ***
1 INYEQFLQALDLIAAQKGMTVERLHEKMLENAAPTNSGTASANSSIVDKLTDSSLYTGAH
2 ANYEQFVHALELIAEQRKTTVEKLHAKLLKAEGPRLSGTIADQGGVLDKLTDSSLYTGAH
*****::**:*** *: ***:** *:*: .* *** : :..::*************

1 KERFDAD**GKGRGLAGR**DSTAKGAGHVGGVHPGGGGVKDLSELLDRSTADVRGVKHDQHDD
2 KHRFNED**GKGRGIAGR**TA--------------NEGVKDMSHVLDRSEADVRGVVPRAE--
*.**: ******:*** : . ****:*.:**** ****** .

1 GKGTMRAAAPAAAATTSARTSSASAPSGGSPKKRPTTSGSASSGGPSGGTVVKGSSDGYDS
2 ---

Salpingoeca dolichothecata **GGOK01021911** Choanoflagellata Craspedida Salpingoecidae
>1
1MTEETKQDKLLHEVFTRFCSVSGLHPIPEMDNTRFVKLCRDVGLIDKKFTRTDADLIFTKCKSKALRKISFEEFQFAMSLVAKKKSKEVTE
LYDAIIETEGPQLMGTKTVEDGVTKRLTDHTQYTGAHRERFDESGKGKGLEGRDQIKKGSGHVRGQATANVHDLSQLTDRSPADVRGVPLHV
KSSSSPKPSPKSERKSSSASLSPPKHKSRSPHTSPKQSPRSSPRSSPSPVRRASTAP240
>2
241KLSSLPPPSSLKDIFQHYCLFGTSGNPDELDGVKFVKMLKDAQLIDAHFSTTEADLIFTKCRAPNLRKVNYEEFRKALECVAEKKALEL
DALEDMLVAAGGPKTGLATKPSDDAIVSKLTDHTQYTGSHKERFDESGRGRGMAGRDLGRKGSGAMPSQGLTYRGGKVDDLSQVTDRGPADS
RGVKYVYKEELEEKK436

1 MTEETKQDKL**L**HEV**F**TR**F**CS**V**SGLHPIPEMDNTRFVKLCRDVGLIDKKFTRTDADLIFTK
2 KLSSLPPPSS**L**KDI**F**QH**Y**CL**F**GTSGNPDELDGVKFVKMLKDAQLIDAHFSTTEADLIFTK
.. . *:::* ::* .. *:*..:***: :*. *** :*: *:*******

1 CKSKALRKISFEEFQFAMSLVAKKKSKEVTELYDAIIETEGPQL-MGTKTVEDGVTKRLT
2 CRAPNLRKVNYEEFRKALECVAEKKALELDALEDMLVAAGGPKTGLATKPSDDAIVSKLT
*:: ***:.:***: *:. **:**: *: * * :: : **: :.** :*.:..:**

1 DHTQYTGAHRERFDES**G**K**G**K**G**LE**GR**DQIKKGSGHVRGQA----TANVHDLSQLTDRSPAD
2 DHTQYTGSHKERFDES**G**R**G**R**G**MA**GR**DLGRKGSGAMPSQGLTYRGGKVDDLSQVTDRGPAD
*******:*:*******:*:*: *** :**** : .*. .:*.****:***.***

1 VRGVPLHVKSSSSPKPSPKSERKSSSASLSPPKHKSRSPHTSPKQSPRSSPRSSPSPVRRASTAP
2 SRGVKYVYKEELEEKK---
*** *.. . *

Chytridiales sp. JEL 0842 **KAJ3407993** Fungi; Chytridiomycota

```
>1
1MANLDDLRVTYNKFCAFGSNRNLSEYSDVQMDGAKFAKFARDTGIIDNKKITTTDIDIIFNKVKPKGARKIDFSAFTAALQMLAEMKYPKK
EPQEALHATLIDVCIKSNGPVAKGTTAQNDAILTRLTDTSLYTGTHKERFDAEGKGKGMAGRDYGSKTDRLDKIVNRDTNATIGISTATFGS
GVILSGSNSGSNSSMNKGRGKYVAGASNERLTGKDFLPKKPAVASDFSHKTQQETTSNNTKKKDTKMATIED 255
>2
256LRVTFNKFCAFGSGSAAENAGMEGSKFAKFARDSGLIDSKKITTTDIDIIFNKVKAKTARKIDFDGFTNAFRMLAEMKYPKKTPEEAFQ
AALEEVVKSSGPVAKGTTAQNDAILNRLTDTSQYTGTHKERFDADGKGKGLAGRDPGPQTDTLDKIVNRETNPTVGLSSKNGITAKSTSSVT
KKSTTNVSSAAPASKLNDLTAKSVSNSKAKLAGTSSVSNSTAKLAKTGSKASIASKSGSKILTDSKRQLNA 507
```

```
1      MANLDDLRVTYNKFCAFGSNRNLSEYSDVQMDGAKFAKFARDTGIIDNKKITTTDIDIIF
2      ------LRVTFNKFCAFGSGSAA---ENAGMEGSKFAKFARDSGLIDSKKITTTDIDIIF
             ****:********.      .:. *:*:********:*:**.************

1      NKVKPKGARKIDFSAFTAALQMLAEMKYPKKEPQEALHATLIDVCIKSNGPVAKGTTAQN
2      NKVKAKTARKIDFDGFTNAFRMLAEMKYPKKTPEEAFQAALEE-VVKSSGPVAKGTTAQN
       **** * ******..** *::********** *:**::*:* :  :**.***********

1      DAILTRLTDTSLYTGTHKERFDAEGKGKGMAGRDYGSKTDRLDKIVNRDTNATIGISTAT
2      DAILNRLTDTSQYTGTHKERFDADGKGKGLAGRDPGPQTDTLDKIVNRETNPTVGLSSKN
       ****.****** ***********:*****:**** * :** *******:** *:*:*: .

1      FGSGVILSGSNSGSNSSMNKGRGKYVAGA-SN---ERLTGKDFLPKKPAVASD--FSHKT
2      GITAKS--------TSSVTKKSTTNVSSAAPASKLNDLTAKSVSNSKAKLAGTSSVSNST
         :.          .**:.*   . *:.*      : **.*..  .*  :*.   .*:.*

1      QQE--TTSN--NTKKKDTKMATIED-----
2      AKLAKTGSKASIASKSGSKILTDSKRQLNA
        :   * *:   :.*..:*:
```

Stylonychia lemnae **CDW85805.1** Ciliophora

```
>1
1MESSNSSLKHVFDSYNGTAKTMEGKNFVKLAKDCKIIDKKLTATDVDLIFAKIKDKSERKITYAQFEKGLEFFAEKKGCTPADIVEKILAT
GGPQFSGTQADHVKFHDDKSLYTGVYAQGGPTNVDQINPTVTFGSAPHNDEEEEKVPTKRMAQ 154
>2
155MSVNNNPAGSLKEVFEGFTGGAAEMDGKTFAKMSKDTKILDKALTATDIDLIFAKVKDKAARKINYAQFQKGIEECATKKKITFEQLEE
KILAVGGPVFTGTKTDKVKFHDDKSLYTGVYAQGGPSTIDAGNGMISDISQLCDRSDANVRGVKKH 309
```

```
1      ---MESSNSSLKHVFDSYNGTAKTMEGKNFVKLAKDCKIIDKKLTATDVDLIFAKIKDKS
2      MSVNNNPAGSLKEVFEGFTGGAAEMDGKTFAKMSKDTKILDKALTATDIDLIFAKVKDKA
           :.  .***.**:.:.* *  *:**.*.*::** **:** *****:******:***:

1      ERKITYAQFEKGLEFFAEKKGCTPADIVEKILATGGPQFSGTQADHVKFHDDKSLYTGVY
2      ARKINYAQFQKGIEECATKKKITFEQLEEKILAVGGPVFTGTKTDKVKFHDDKSLYTGVY
        ***.****:**:*  * **  *  :: *****.*** *:**::*:**************
1      AQGGPTNVDQINPTVTFGSAPHNDEEEEKVPTKRMAQ
2      AQGGPSTIDAGNGMISDISQLCDRSDANVRGVKKH--
       *****:.:*  *  ::  *   : .: :   .*:
```

Halteria grandinella **TNV76936.1** Ciliophora

```
>1
1MQSAEPIQPPQTLGDQLNSVFEAYAIDRIHLEGKQFAKIFRDCSLIDKKLTQPEIDIAFAKAKQIKTERKLNFEEFLLALDFCAQKKGIPT
ETLAYELVARCQGPLYVAITDQVRLHDDKTTYTGVYAQGGPRTVDGSPKVTISKLGLEEFKNDYDKSPK 161
>2
162VSFVAAQPASTLEEVYAGFTFSQPDMDGKTFVKLFKDCKILDKKLTTTDLDILFSKIKAKGQRKIGFEQFLTALQEISQLKSITFDQLK
NQICSVGGPVFTGTRAEKVKWHDDKSTYTGVYAKGGPETVDIGRTMINDIRHLCDRSEADIRGVKKH 307
1      MQSAEPIQPPQTLGDQLNSVFEAYAIDRIHLEGKQFAKIFRDCSLIDKKLTQPEIDIAFA
2      -VSFVAAQP----ASTLEEVYAGFTFSQPDMDGKTFVKLFKDCKILDKKLTTTDLDILFS
         *    **    .. *:.*: .:::.: .::** *.*:*:**.::*****  ::** *:

1      KAKQIKTERKLNFEEFLLALDFCAQKKGIPTETLAYELVARCQGPLYVATITDQVRLHDD
2      KIK-AKGQRKIGFEQFLTALQEISQLKSITFDQLKNQ-ICSVGGPVFTGTRAEKVKWHDD
       * *  * :**:.**:** **:  :* *.*  : *  : :.   **::..* :::*: ***

1      KTTYTGVYAQGGPRTVDGSPKVTISKLGLEEFKNDYDKSPK--------
2      KSTYTGVYAKGGPETVDIGRTMIN------DIRHLCDRSEADIRGVKKH
       *:*******:***.*** . .:        ::::  *:*
```

Tunicaraptor unikontum **GIQG01040147.1, GIQG01040148.1**
>1
1MSYKEAEMKQLFEAFVSFGAGSNGPAAMEMDSAKLTKFCKENSVFNKKCTSTDSDIIFSKVKTKGARKIVFSEFVAALQLLAEKRGSAYED
VVDKAIEKGGPASSGTKAQPNKFYDDKSNWTGTAAKGGPSTVDLANQDLSKHLDRTDADVRGVKKAATTTARPTSAGGSSARPPSAKGARPV
SATKDSVKSPRPGSRPGSAVKRPGSSASSKNTRLD 218
>2
219PETTEGQLAELFTTFCAFGGGVQPSYLSAEMDGAKFVKFCKDSKLVGKKMTVTDVDLIFTKSKFDKTDRKITWDSFRQAALPQMAEKLG
TSVEQVMEKAVAAGGPTSSGTKAQSNRFHDDKSLYTGTHAKGGPSTVDHANQDLSAHLDRAPADVRGVKK 377

```
1       MSYKEAEMKQLFEAFVSFGAGSNGP-AAMEMDSAKLTKFCKENSVFNKKCTSTDSDIIFS
2       PETTEGQLAELFTTFCAFGGGVQPSYLSAEMDGAKFVKFCKDSKLVGKKMTVTDVDLIFT
            *.:: :** :* :**.* :    : ***.**:.****:..:..** * ** *:**:

1       KVKTK-GARKIVFSEFV-AALQLLAEKRGSAYEDVVDKAIEKGGPASSGTKAQPNKFYDD
2       KSKFDKTDRKITWDSFRQAALPQMAEKLGTSVEQVMEKAVAAGGPTSSGTKAQSNRFHDD
        * * .   ***.:..*  ***  :*** *:: *:*::**:  ***:******* *:*:**

1       KSNWTGTAAKGGPSTVDLANQDLSKHLDRTDADVRGVKK
2       KSLYTGTHAKGGPSTVDHANQDLSAHLDRAPADVRGVKK
        ** :*** ********* ****** ****: ********

1       AATTTARPTSAGGSSARPPSAKGARPVSATKDSVKSPRPGSRPGSAVKRPGSSASSKNTRLD
```

Savillea parva **GGOL01030293.1** Choanoflagellata Acanthoecida; Acanthoecidae
>1
MPLSAEDEFKLLDVFCSFCGADAEKGQMDGRQFAKMTRDCKMLDDSFTSIDTDIIFAKCKAKAMRKIDFHQFLDAINMCAATKRVSPSALAA
KMVKAGGPKIGGTHAGKVRLHDDKAGYTGVAANGGPVTVDTGRAPSMGKPSTPREHAHHVANVTATLH 150
>2
151ATHVVEGEYTLQTFFKDHSGGAKEMDGRQFAKMARDTKLIDKKFTSIDLDIIFAKVKAKASRKITFKQFKDAITLCAEKKGKSFEELED
YLLKSGGPVFTGVKTDKVKLHDDKSTYTGVYTHGGPDTKSLIAHHDITNLTDRTQADVRGVKRH 303

```
1       MPLSAEDEFKLLDVFCSFCGADAEKGQMDGRQFAKMTRDCKMLDDSFTSIDTDIIFAKCK
2       ATHVVEGEYTLQTFFKD---HSGGAKEMDGRQFAKMARDTKLIDKKFTSIDLDIIFAKVK
            .*.*:.*  .* .    ..   :*********:** *::*..***** ****** *

1       AKAMRKIDFHQFLDAINMCAATKRVSPSALAAKMVKAGGPKIGGTHAGKVRLHDDKAGYT
2       AKASRKITFKQFKDAITLCAEKKGKSFEELEDYLLKSGGPVFTGVKTDKVKLHDDKSTYT
        *** *** *:** ***.:** .*  * . *   ::*:*** : *.::.**:*****: **
1       GVAANGGPVTVDTGRAPSMGKPSTPREHAHHVANVTATLH
2       GVYTHGGPDTKSLIAHHDITNLTDR-TQ-ADVRGVKRH--
        ** ::*** * .     .: : :    :  .* .*.
```

Stephanoeca diplocostata **GGOM01054003.1** Choanoflagellata Acanthoecida; Stephanoecidae
>1
1MASVIDDDGLVEMFCLFAGVQGTETGLMDGRQFVKLLRDTNLVTKKFTATDVDIIFAKAKEKGMRKITYDQFCDALRMVAEKKKMTFESLV
LKLVTAEGPVLKGTSAQKVALHDDKSLYTGVYANGGPSTVDSDRTVDFGKQMGRDTADVRGVVIGGFDTTPHLSKTHAAADGGEGVTHAASP
TKNSPRTVQAPPPTAVARPS 203
>2
204SAETATSGTLEGVFSEHSLGAKEMDGRQFVKLCKDSNLLSKKKFTSTDVDIIFAKAKGKGSRKLTFPQFEHAIAEIATALRVDASEVVA
RLVANGGPVFSGVRTDAVRLHDDTDGYTGVYKQGGPAVTGDHGPRVDSIATLCDRSPADVRGVKQ 357

```
1       MASVIDDDGLVEMFCLFAGVQGTETGLMDGRQFVKLLRDTNLVT-KKFTATDVDIIFAKA
2       SAETATSGTLEGVF----SEHSLGAKEMDGRQFVKLCKDSNLLSKKKFTSTDVDIIFAKA
         *..  .. *  :*    . :.  :  ********* :*:**:: ****:**********
1       KEKGMRKITYDQFCDALRMVAEKKKMTFESLVLKLVTAEGPVLKGTSAQKVALHDDKSLY
2       KGKGSRKLTFPQFEHAIAEIATALRVDASEVVARLVANGGPVFSGVRTDAVRLHDDTDGY
        * ** **:*: ** .*:  :*   ::  ..:* :**:  ***:.*. :: * ****.. *

1       TGVYANGGPSTVDSDR—TVDFGKQMGRDTADVRGVVIG
2       TGVYKQGGPAVTGDHGPRVDSIATLCDRSPADVRGVKQ
        **** :***:.....   . .:..  .*. ******

1       GFDTTPHLSKTHAAADGGEGVTHAASPTKNSPRTVQAPPPTAVARPS
```

Diaphanoeca grandis **GGPB01024076.1** Choanoflagellata Acanthoecida Stephanoecidae

```
>1
1MAATIIFSQFTGDAHGGAKKAQMDGRQFSKMAKDCVIKDGETAWSLLDKKFTSTDIDIIFSKVKAKGERKINVVEFTNALTEISVKKNMTF
AELCERIMESGGQKFTGTKATHVKLHDDKAGYTGVSSRGGPSTVDELNVADLSVFTERTPHGPGKGLQGSPSPKKSPKQHRNVETISTS180
>2
181AAAESLEQVFLGFTNGAKEMDGRQLAKLTKDAKILNKKITATDVDISFAKFKSKGARKINYTQFEQVIESFALKEKITKEVLVERILAK
GGPTFSGTKTQGSRLHDDKSTYTATQAHGGPTLVGRGFRGEGEVDDLSQLADRTSADVRGTKH332
```

```
1       MAATI----IFSQFTGDAHGGAKKAQMDGRQFSKMAKDCVIKDGETAWSLLDKKFTSTDIDIIF
2       SAAAESLEQVFLGFTN----GAK--EMDGRQLAKLTKDA---------KILNKKITATDVDISF
         **:     :*  **     ***  :*****::*::**.         .:*:**:*:**:** *

1       SKVKAKGERKINVVEFTNALTEISVKKNMTFAELCERIMESGGQKFTGTKATHVKLHDDK
2       AKFKSKGARKINYTQFEQVIESFALKEKITKEVLVERILAKGGPTFSGTKTQGSRLHDDK
        :*.*:** **** .:* :.: .:::*:::*   * ***: .** .*:***:   :*****
1       AGYTGVSSRGGPSTVDEL-----NVADLSVFTERTPHGPGKGLQGSPSPKKSPKQHRNVETIST
2       STYTATQAHGGPTLVGRGFRGEGEVDDLSQLADRTSADV----RGTKH----------------
        : **...::***: *..      :* *** :::**  .     :*:
```

Helgoeca nana **GGOR01010129.1** Choanoflagellata Acanthoecida Acanthoecidae

```
>1
1MSGFTPDEETALLDVFCAFCGVDAEKGQMDGRQFAKMTRDTNLIDKKFTSIDTDIIFAKVKAKSLRKIDFHQFLSAIEMCATSKRVPVSDV
VAALVTGGGPKMTGKSPARSTKVRLHDDKAGYTGVHARGGPSTVDGERHIDFGEPSPRAPSAKVSTVTASLQ 163
>2
164ETHITEGGEYTLKMFFTDHAGGGKDMDGRQFAKMAKDTKLIDKKPPGLTTIDLDIIFAKVKDKSARKITFKQFREALKACAEKKHMYQD
ALEEHLVASGGPVFTGVKTEKVRLHDDKAAYTGVYSHGGPDTGSLTAHHDISTLTDRSQADVRGVKR 319
```

```
1       MSGFTPDEETALLDVFCAFCGVDAEKGQMDGRQFAKMTRDTNLIDKK---FTSIDTDIIF
2       ETHITEGGEYTL-KMF--FTDHAGGGKDMDGRQFAKMAKDTKLIDKKPPGLTTIDLDIIF
         : :* . * :* ::*  * .  .   :*********::**:*****   :*:** ****

1       AKVKAKSLRKIDFHQFLSAIEMCATSKRVPVSDVVAALVTGGGPKMTGKSPARSTKVRLH
2       AKVKDKSARKITFKQFREALKACAEKKHMYQDALEEHLVASGGPVFTGV---KTEKVRLH
        **** ** *** *:** .*:: ** .*::  . :   **:.*** :**    :: *****

1       DDKAGYTGVHARGGPSTVDGERHIDFGEPSPRAPSAKVSTVTASLQ
2       DDKAAYTGVYSHGGPDTGSLTAHHDISTLTDRSQAD-VRGVKR---
        ****.****:::***.* .   * *:.  : *: :  *  *.
```

Acanthoeca spectabilis **GGPA01008943.1** Choanoflagellata Acanthoecida Acanthoecidae

```
>1
1MASSFSAEEESRLLEVFCLFCGVDAEKGQMDGRQFAKLMRDTGMLTKKFTSIDTDIIFAKVKAKALRKIDFNQFLAAAELCAGAKKVPVSD
VVATLTSGGGPKVAGTRTDKVRLHDDRGGYTGVHSKGGPSTVDSERQIDFGTPPPRAAKASSGVASATSQMQSMQVSDGGYT173
>2
174MQSMQVSDGGYTLKMFFTDHSLGAKEMDGRQFVKMAKDTKIVDKKPPGLTTTDLDIIFAKVKAKSARKINFAQFQEALVHCAEKKHVYL
EDLEAHLVAFGGPVFTGVKADNVKLHDDKSTYTGVYTHGGPDTGSMGAHHDISTLTDRTPADVRGVKR330
```

```
1       MASSFSAEEESRLLEVFCLFCGVDAEKGQMDGRQFAKLMRDTGMLTKK---FTSIDTDII
2       -MQSMQVSDGGYTLKMF--FTDHSLGAKEMDGRQFVKMAKDTKIVDKKPPGLTTTDLDII
          .*:...: .  * :*  * . .    :******.*: :** :: **   :*: * ***

1       FAKVKAKALRKIDFNQFLAAAELCAGAKKVPVSDVVATLTSGGGPKVAGTRTDKVRLHDD
2       FAKVKAKSARKINFAQFQEALVHCAEKKHVYLEDLEAHLVAFGGPVFTGVKADNVKLHDD
        *******: ***:* **  *   **  *:* :.*: * *.: *** .:*.::*:*:****
1       RGGYTGVHSKGGPSTVDSERQIDFGTPPPRAAKASSGVASATSQ
2       KSTYTGVYTHGGPDTGSMGAHHDISTLTDRTPADVRG-------
        :. ****:::***.* .   : *:.*   *:     *
```

Figure S2: Newly identified p25alpha domain containing proteins in class Filasterea

>Mvib_g11715 *Ministeria vibrans* **Long p25alpha domain containing protein**

MPDTEKPESVAAAAEEASEAAVASAGPTSVVAETTSAAAAKEEKAPSRPTSAVAKTEKVASRPTSGKAKGNLTPAGLRPTSSKTKPESASEP
AAALIATEEANPATPNPAVTKVSKPSTPKAVSKPSTPKPSAPKAVSKPSTPKPSTPKAVSKPSTPKPSTPQPSAPKPSTPKAVSKPSTPKAV
SKPSTPKPSTPKAVSKPSTPKPSTPKAVSKPSTPKPSTPQPSASKPSTPKAVSKPSTPKPSTPQPSASKPSTPKAVSKPSTPKQSTSKPSTA
APATATATTTTTPKSSSKPSTPKPSSIPRVSSPLVPKKDQPLKSTSPAPQLSGTALDSA**L**ETS**F**TA**Y**VT**F**GGGKDSVKMTGKQFDKWAKEAK

```
VIGINGVTSTDTDIAFSKVARGQKPVGLDGIREIVLEIARKVGKAEAKKKKEGEETLIAHWNEKIIHKLANAMPKLQGTTKVSNVGNVEGLT
DTAKYTGTHKERFDKDGKGKGKVGREDIVKSKGFVGAYKGEGTYDKTH

>Pchi_g246 Pigoraptor chileana Apicortin
MSDAEARPASSSSLKRAVEAAKSGSTASLKAAAAADATPARPASSSSLKKAVEAAKSGSTASLKTAVAADKAAGSTASLKGAAAAEKAGSQA
SLKTSPAKEKSPAKAKGQSVTDRLTDASKFTGAHKERFDESGKGKGLAGREELVNVDGSTSSAARDHTVKSSTAAAAEKKPVVSGNLGKEKF
GTQADKPISITLFRNGDKNHAGEKIVPAKFKMFRTMDQLHKEATDIVKLVTGPVRKIYKADCKTQITALDQFENGGKYLCCSGEAPAPADKL
PAALTA

>Pvie_g6368 Pigoraptor vietnamica Apicortin
MSTPAKKPATTAGTKAAAPKAKAASVTDRLTDTSKYTGAHKERFDDSGKGKGIAGRENVVKVDGSTESASRDHAVQSTAVEKSAKPVVSGNL
GSQKFGTQADKPISIILFRNGDKNHTGEKIPPAKFKTLRSMDQLRKEATDIVKLVTGPVRKIYKTDCKTQITALDQFEDGGKYLCCSGEAPA
AADKLPSAFTS
```

Color code: grey - PHA03247 domain, yellow – partial p25alpha domain, blue – DCX domain. Sequences are from Reference [22]. (doi: 10.1038/s41586-022-05110-4).

Figure S3: Multiple alignment of long type TPPPs

```
#NEXUS
[TITLE: Written by EMBOSS 22/10/25]

begin data;
dimensions ntax=35 nchar=245;
format interleave datatype=protein missing=X gap=-;

matrix
Tetrahymena           LEGVFKKFTA-----------NKADMDGKTFAKFAKDCGL-LDKKLTATD
Ministeria            LETSFTAYVTFGGGKD------SVKMTGKQFDKWAKEAKVIGINGVTSTD
Symsagittifera1       lqsvfnefcsfgagtgk----tkteldsknfkkmtedcglfkkgkldgae
Symsagittifera3       lksaflafsnfgagank----qknemesknfkklvqecglfkkgkldgae
Bolinopsis1           meetfanfskfgdsks-----tgkeldnkkfsklckdsgi-mgklvtstd
Bolinopsis2           meetfanfskfgdtks-----tgkeldnqkfsklckdsgi-mgklvtstd
Beroe                 MEETFANFSKFGDTKS-----TGKELDNKKFSKLCKETGI-MGKLVTSTD
Mnemiopsis1           meatftafskfgdsks-----tgkeldnkkfsklckdtgi-mgklvtstd
Mnemiopsis2           meetfanfskfgdtks-----tgkeldnkkfsklckdtgi-mgklvtstd
Suberites             lestfngfcsfgaskd-----gaalmdnakfaklfrdlkl-ldkkftstd
Amphimedon            ledvfqsfcsfgeglk-----gsaamdnakfakltrdvki-ldkkltstd
Corticium             medifksfcafgsgke-----stalmdnakfakfardtki-ldkkltstd
Oscarella             letvfksfcafgagk-d----aislmdnakfskffrdlkl-ytkkftqtd
Nematostella1         LQAKFESFCAFGAGAKG----AQPLMDNAKFGKMFRDLHL-YDQKFTSTD
Nematostella2         LHEIFKSFCAFGAGCKD----AQPLMDNAKFAKLFRDLKI-YDKKFTQID
Hydra                 mesvfksfcafgsrkde----tvdlmdnakfsklardlki-ldkkltstd
Rhopilema             metlfksycsfgak-d-----aaplmdnakfakfardtki-ldkkltatd
Amoeboaphelidium      vrqtfeqfanfgsssae----khetidnakfaklcrdcgi-idkkctsvd
Choanoeca             LEAVFQSFASFGAGSA-----GAAELDSSKFSKLCKECKL-FTKSFTTTD
Mylnosiga             LQEAFARFASFGAGKDS----SAAELEGAKFAKLCKDCKL-VAGGFTTTD
Monosiga              LKQVFEQYASFGAGSAA---TGTPELDSAKFTKLCKETKL-ISKSLTTTD
Hormiphora            MEEVFAAFSKFGDTKS-----TGKELDNKKFSKLCKDTGI-MGKLVTSTD
Chytriomycesc         lkevfekf---ga---------kdgemdgtkfakftkdtglmdgkkvttte
Obelidium             LREVFDKF---G---------KDGEMDGAKFAKFTKDAGLVDGKKITTTE
Globomyces            LNRAFVAYCQFGASKSN--PSDALTMDGARFAKFCKDTGV-IGGPITNTD
GorgonomycesA         LIEVFESYCSFGSSKTLSMNSMGGQMDGAKFAKLCKDKKL-ISGKITSTD
GorgonomycesB         VEEIYNRFCTFGSRTLN--QDLGTWMDNSKFAKLCRDTKI-VGGKITPTD
Chytriomycesh         LKEVFEKF---GA--------KDGEMDGTKFAKFTKDTGLMDGKKVTTTE
Sanchytrium           LLKRFSAFCSFGSSRAI----NAVEIDNFKFSKLCKDSLI-LDKKLTTTD
Amoeboradix           LRDRFNAFCSFGSSRAV----VSVEIDNSKFSKLARETGI-IDKKCTSTD
Homo                  LEESFRKFAIHGDPKA-----SGQEMNGKNWAKLCKDCKVADGKSVTGTD
Drosophila            FSDQFKAFSKFGDSKS-----DGKLITLSQSDKWMKQAKV-IDKKITTTD
Caenorhabditis        VKKRWDAFTKFGAAT--------ATEMTGKNFDKWLKDAGVLDNKAITGTM
Strongylocentrotus1   lqdvfksfcafgagskd----aapvmdnskwgkmfrdlkl-ydkkftstd
Strongylocentrotus2   lkgvfqkyavfgrpgq------tkditsknfskmmkecdi-mdkkvnqte

Tetrahymena           IDLIFAKV-KTSSAVRTITFAQFEK-GLDQMAT--------KKGIS----
Ministeria            TDIAFSKVA---RGQKPVGLDGIRE-IVLEIARKVGKAEAKKKKEGEETL
```

```
Symsagittifera1       mdmiftrc-knp--arhipfdkfkaqavpqlak--------avygadsee
Symsagittifera3       vdmiftrc-kgpmpcrnilfdsfkdkaipqlat--------avygddsee
Bolinopsis1           vdiifsk--ykpkggrtitlkefnl-ildelcv--------krfpkketl
Bolinopsis2           vdiifsk--ykpkggrtitlkefnl-ilddlcv--------krfpkketl
Beroe                 VDIIFSK--YKPKGGRTITIKEFNL-ILDELCV--------KRFPKKESL
Mnemiopsis1           vdicfsk--hkpkggrtitlkefka-iledlsv--------krfpkhs-p
Mnemiopsis2           vdicfsk--hkpkggrtitlkefea-ilddlcv--------krfpkketl
Suberites             vdiifnrpevkakgerkinfaqfqa-alklvae--------kkypgds--
Amphimedon            vdiifsk--vkaktdrkinfeqfke-avrlmad--------kkypgdp--
Corticium             vdiifnk--vkgkserkitfdqfre-alkllae--------kkypgda--
Oscarella             adiifsrpevkskterkinfkqftk-alelsae--------kmg------
Nematostella1         TDIIFSRTEVKPKTERKINFNQFKV-ALGLCAE--------KKFG-SK--
Nematostella2         SDIIFNRPEVKSKSERKVNFSQFKA-ALSLCAE--------KKYG-KR--
Hydra                 vdiifnk--vkskterkityhqfed-gvklmae--------kkypgda--
Rhopilema             vdiifsk--vkakterkitykqfqd-alkllae--------kkygep---
Amoeboaphelidium      vdiifkk--vvrkgerridfaefqk-alkqlse--------kkfradd--
Choanoeca             ADIIFTK--AKTKGKRKITFAEFEQ-ALDMVAE--------KKGKS----
Mylnosiga             VDLIFAQ--CHAKGARKIDFAGFKK-ALQLIAD--------KKKIA----
Monosiga              ADLIFTR--VKAKGQRKIGFAEFRS-ALEEVAK--------KTGQD----
Hormiphora            IDIIFSK--YKPKGGRTITIKEFGA-ILDELAK--------KRFPKKGSP
Chytriomycesc         vdivfgk--vkaktarkidlaqfes-aigllad--------krypgkshe
Obelidium             VDIVFNK--AKAKTARKIDFAAFES-ALGMLAD--------KRYPGKPHE
Globomyces            VDIVFSK--VKAKNARRINQQEFIL-GIQQLGE--------KKFKGTP-E
GorgonomycesA         IDIIFNK--VKEKTARKIGFPEFME-ALKLIAA--------KKYEDKSQN
GorgonomycesB         VDIIFNK--VRTKNPRKIDFDEFLQ-GLKLLGA--------IKFPEKEPL
Chytriomycesh         VDIVFGK--VKAKTARKIDFAQFET-AIGLLAD--------KRYPGKSHE
Sanchytrium           VDIVFNKA-KKEKTDRKLDFEQFKA-ALRLLAE--------IKYPGDE--
Amoeboradix           VDIIFNKA-KKNKTDRKLDFDQFKV-ALKLLAE--------VKYPGE---
Homo                  VDIVFSK--VKGKSARVINYEEFKK-ALEELAT--------KRFQGKS-K
Drosophila            TGIHFKK--FKA---MKISLSDYNK-FLDDLAK--------TKKVE----
Caenorhabditis        TGIAFSKVT---GPKKKATFDETKK-VLAFVAEDRARQ---SKKPIQ---
Strongylocentrotus1   tdiifnrpevksktdrkinfaqfkk-alelcae--------kkyg-sk--
Strongylocentrotus2   idiifqraka-spklkvltyekflt-slkmiak--------skygtdeee

Tetrahymena           ----LDALKEKVTSA--GGPT-------------------FTGTK---A
Ministeria            IAHWNEKIIHKLANA---MPK------------------LQGTTKVSNV
Symsagittifera1       ---nqkkiidaitsn---spk------------------nktsakasnd
Symsagittifera3       ---nkkqiidaitsn---spk------------------vnssvkmcsi
Bolinopsis1           d--ekkgairtliag--kgpk------------------tkgttkvskk
Bolinopsis2           d--ekkeairtliag--kgpt------------------tkgttkvstk
Beroe                 D--DKKQAIRELIAg--kgpg------------------ttgttkLANK
Mnemiopsis1           t--erksameelvsn--kgpq------------------tagttkavkg
Mnemiopsis2           d--ekkeairtliag--kgpk------------------tagttkvskk
Suberites             d--glkkltdkiltg--kgpa------------------tsgatkfkss
Amphimedon            d--gerklidkitag--sgpk------------------vqgvtktvds
Corticium             d--gakkledfvlqg--kgpt------------------gnkttsgvkt
Oscarella             i--dyasltdkivag--kgpv------------------avgatkatka
Nematostella1         D--QVGKLTEKICKG--KGPA------------------TSGATKAVKV
Nematostella2         E--DVIKLVDKICEG--KGPV------------------ASKTTKVVKA
Hydra                 e--gynklkdlinsg--sgpt------------------asgvtktaks
Rhopilema             ---nvekleevilkt--sgpk------------------asgvtkavka
Amoeboaphelidium      g--gyqkvlalvaks---aps-------------------tsgtltead
Choanoeca             ----KAAIVEQILQA--GGPM-------------------SSGTRPSSG
Mylnosiga             ----LADVEAAI-VT--HSPS-------------------SSGTHAVDS
Monosiga              ----VSAVEAKVTRA--GGPQ-------------------SSGTQADSG
Hormiphora            E--EQKAAIRELIQG--KGPG------------------TSGTTKTVKA
Chytriomycesc         e--svasamsdlcka--kgpv-------------------ikgtkaqed
Obelidium             E--ALANTMADVCKT--KGPI-------------------LKGTVAQND
Globomyces            D--SLNNIISQILKSG-GVPK-----------------LAEGATQVEKS
GorgonomycesA         D--ALGTLINDMLTNTSSLPS------------------LNKTTKASTS
GorgonomycesB         R--QFHLMCDIVVNGKPK----------------------VKATSVQSD
Chytriomycesh         E--SVASAMSDLCKA--KGPV-------------------IKGTKAQED
Sanchytrium           E--GYSKLVALVVKNEPKVRTETGSNKNLNGS-------GNELDSKASSD
Amoeboradix           D--GYLKIVQLVNTSEPKVRKEVGSSGNLKSISLSDVRSESESNTKQSSD
Homo                  E--EAFDAICQLVAG--KEPA------------------NVGVTKAKTG
Drosophila            ----LSEIKQKLASC--GAPG-------------------VVSVSAGKAA
Caenorhabditis        D--ELDAITEKLAKL--EAPS-------------------VGGAAKANAA
```

```
Strongylocentrotus1  d--dvqkliekicag--kgpg-------------------tsgatkaska
Strongylocentrotus2  ---nfgkiknqirss--sgps-------------------tagttststt

Tetrahymena          DAVKFHDDKSLYTGVYANG----GPS------TVDIGNGKISDISQLCDR
Ministeria           GNVEGLTDTAKYTGTHKERFDKDGKGKGKVG-REDIVK------------
Symsagittifera1      kvvgrltdtskytgahkerfdesgkgkgadg-redkaa------------
Symsagittifera3      gvvdrltdpskytgah----------------------------------
Bolinopsis1          ggtdrltdtskytgshkerfgtdgkgkgleg-redvie------------
Bolinopsis2          gvtdrltdtsrytgshkerfgtdgkgkglkgtredtle------------
Beroe                EATARLTDTSKYTGSHKERFGADgkgkgieg-reerve------------
Mnemiopsis1          gatgrltdtskytgshkerfgedgkgkgleg-rkelad------------
Mnemiopsis2          ggterltdtskytgshkerfgadgkgkgleg-redvie------------
Suberites            gavdrltdtskytgshkerfdesgkgkgleg-rdtgakghgmaagsvag-
Amphimedon           pllermtdtskytgthkerfdesgkgkglag-rdsfqkgagmapdgfsg-
Corticium            ggvdrltdtskytgshkqrfdesgkgkgleg-rqdfdd---------ka-
Oscarella            gavdrltdtskytgshkerfdesgkgkgldg-rydvdd---------ka-
Nematostella1        GGVERLTDTKCYTGSHKERFDKSGKGKGIEG-RVDRDD---------KA-
Nematostella2        GAVDRLTDTRKYTGSHKERFDETGKGRGIEG-RVDRDP---------HA-
Hydra                dtverltdtskytgshkerfdesgkgkgldg-rrefde---------ka-
Rhopilema            ggvdrltdtskytgshkerfdesgkgkgleg-rkdfde---------ka-
Amoeboaphelidium     gvfekltdtslytgthkekfdkdgnakplgn-gin----ktadlsqivar
Choanoeca            GVVNRMTDTAQYTGSHKHRFNEDGKGRGLAG-RDTITKGTGHIPAAI---
Mylnosiga            GIVKKLTDTTLYTGSHKERFDDDGKGKGLSG-RDPAAKGGGHLPPGAHTH
Monosiga             GVLDRMTDTSQYTGSHKERFDSEGHGKGLAG-RDSTAKGTGHIPAV----
Hormiphora           GGTERLTDTSKYTGSHKERFDESGKGKGLGG-REELVE------------
Chytriomycesc        evtkrmtdvsgytgthkerfnedgtgkglag-rdqpvqtad--lsqiva-
Obelidium            EVTKRMTDVSQYTGTHVHRFNEDGTGKGKAG-RDAPSSTAD--LSQIVA-
Globomyces           TIVEKLTDTSKYTGTHKERFDGNGKGKGLDG-RTDLGSQTL-SLSQITNR
GorgonomycesA        DLTKRLTDTSQYTGTHKQRFDESGQGRGKAG-RDDGSQ---ITLQSIANR
GorgonomycesB        PVTQRLTDTSQYTGTSKNRFDDNGKGLGMTLLGSQANL------SKLSRD
Chytriomycesh        EVTKRMTDVSGYTGTHKERFNEDGTGKGLAG-RDQPVQTAD--LSQIVA-
Sanchytrium          SVFDRLTDHTKYTGSHKERFDDDGKGKGLDG-RRDGQVNHISE---ILRK
Amoeboradix          SVFDRLTDHTKYTGSHKERFDEDGKGKGLEG-RTTGEVHHISQ---ILRK
Homo                 GAVDRLTDTSRYTGSHKERFDESGKGKGIAG-RQDILD------------
Drosophila           AAVDRLTDTSKYTGSHKERFDASGKGKGIAG-RRNVVD------------
Caenorhabditis       GVYSRLTDHTKYTGAHKERFDAEGKGKGKSG-RADTTE------------
Strongylocentrotus1  ggvdrltdsskytgshkerfdesgkgkgldg-rkdfda---------ka-
Strongylocentrotus2  gkvdhftdvtkytgqhrerfekdgtgkgkag-reylve------------

Tetrahymena          TG---------------------ADVRGVKK--------------
Ministeria           ---------------------SKGFVGAYKGEGTYDK-TH-----
Symsagittifera1      ---------------------ndgyvqgyqnkgtkk---------
Symsagittifera3      ---------------------------------------------
Bolinopsis1          ---------------------ddgyvvgytnkdtyek-dhedn--
Bolinopsis2          ---------------------ydgyvvgyknkdtyge-ah-----
Beroe                ---------------------NDGYVAGYKNNDTWDK-IRD----
Mnemiopsis1          ---------------------dsgyvgnykgaetydk-th-----
Mnemiopsis2          ---------------------ddgyvvgytnkdtyde-th-----
Suberites            ---------------------qagyvsgykhegtydk-kk-----
Amphimedon           ---------------------nasyvhgykhegtydk-kvkk---
Corticium            ---------------------asgyvggykgqgtyds-kk-----
Oscarella            ---------------------ssgyvggykgkdtydk-sh-----
Nematostella1        ---------------------AQGYVGNYKGEGTYDK-TH-----
Nematostella2        ---------------------EAGYVGNYRGRDTYDQ-TH-----
Hydra                ---------------------sagyvggykemntydq-nhk----
Rhopilema            ---------------------asgyvggykgkdtydg--------
Amoeboaphelidium     ks-gttpver----------stakstgatkaggknpk-asgq-ra
Choanoeca            -APGTSADLASHLDRSP------ADVRGVK---------------
Mylnosiga            VGGGGFHDLSSITDRSA------ADVRGVPVRASEAKKSPAPTRA
Monosiga             ---GGGSDLASHLDRSP------ANVRGVKK--------------
Hormiphora           ---------------------NTGYVGNYKGADTYDK-SH-----
Chytriomycesc        ---------------------------nk----------------
Obelidium            ---------------------------NK----------------
Globomyces           K----EADVR-------------GVAK------------------
GorgonomycesA        ---DDRAD-------------VRGVNQSFKK--------------
GorgonomycesB        NL-NKRAESKEMLQEGPTKLPKIGSKGNLTRA-------------
Chytriomycesh        ---------------------------NK----------------
```

```
Sanchytrium         ------------------------------------------
Amoeboradix         ------------------------------------------
Homo                --------------------DSGYVSAYKNAGTYDA-KVKK---
Drosophila          --------------------GSGYVSGYQHKDTYDN-AH-----
Caenorhabditis      --------------------NTGYVGAYKNKDSYDK-THGK---
Strongylocentrotus1 --------------------aegyvggykgkdtydk-k------
Strongylocentrotus2 --------------------esgyvtgykgkdsfdg-ke-----
;

end;
begin assumptions;
options deftype=unord;
end;
```

Figure S4: Bayesian tree for Fig. 3

```
#NEXUS

[ID: 2565083671]
begin trees;
   [Note: This tree contains information on the topology,
          branch lengths (if present), and the probability
          of the partition indicated by the branch.]
   tree con_50_majrule =
(Tetrahymena:0.714304,((((Ministeria:0.561616,Drosophila:0.468097,Caenorhabditis:0.438179)1.
00:0.208313,((((((Bolinopsis1:0.030982,Bolinopsis2:0.073641)0.89:0.032302,Mnemiopsis2:0.0359
15)1.00:0.065823,Beroe:0.097739)1.00:0.097697,Mnemiopsis1:0.157022)0.55:0.037279,Hormiphora:
0.086081)1.00:0.269422,Homo:0.346316)0.59:0.098984)0.98:0.149435,((Symsagittifera1:0.112535,
Symsagittifera3:0.240031)1.00:0.440881,Strongylocentrotus2:0.669326)0.97:0.184976)0.53:0.070
509,((Suberites:0.150425,((Corticium:0.160776,(Hydra:0.211534,Rhopilema:0.146158)0.71:0.0486
27)1.00:0.082028,(Oscarella:0.182183,((Nematostella1:0.159192,Nematostella2:0.207673)1.00:0.
074136,Strongylocentrotus1:0.079270)0.97:0.061925)1.00:0.108161)1.00:0.103081)0.52:0.057905,
Amphimedon:0.277740)1.00:0.131580,((Amoeboaphelidium:0.512166,(Sanchytrium:0.184323,Amoebora
dix:0.131415)1.00:0.242755)1.00:0.217368,((((Chytriomycesc:0.012436,Chytriomycesh:0.009188)1
.00:0.093285,Obelidium:0.116305)1.00:0.330428,(GorgonomycesA:0.333568,GorgonomycesB:0.707554
)0.77:0.107599)0.74:0.138292,Globomyces:0.490704)0.84:0.132253)0.50:0.124842)1.00:0.227266,(
(Choanoeca:0.174211,Monosiga:0.236119)0.99:0.128606,Mylnosiga:0.465764)0.90:0.123734);

   [Note: This tree contains information only on the topology
          and branch lengths (mean of the posterior probability density).]
   tree con_50_majrule =
(Tetrahymena:0.714304,((((Ministeria:0.561616,Drosophila:0.468097,Caenorhabditis:0.438179):0
.208313,((((((Bolinopsis1:0.030982,Bolinopsis2:0.073641):0.032302,Mnemiopsis2:0.035915):0.06
5823,Beroe:0.097739):0.097697,Mnemiopsis1:0.157022):0.037279,Hormiphora:0.086081):0.269422,H
omo:0.346316):0.098984):0.149435,((Symsagittifera1:0.112535,Symsagittifera3:0.240031):0.4408
81,Strongylocentrotus2:0.669326):0.184976):0.070509,((Suberites:0.150425,((Corticium:0.16077
6,(Hydra:0.211534,Rhopilema:0.146158):0.048627):0.082028,(Oscarella:0.182183,((Nematostella1
:0.159192,Nematostella2:0.207673):0.074136,Strongylocentrotus1:0.079270):0.061925):0.108161)
:0.103081):0.057905,Amphimedon:0.277740):0.131580,((Amoeboaphelidium:0.512166,(Sanchytrium:0
.184323,Amoeboradix:0.131415):0.242755):0.217368,((((Chytriomycesc:0.012436,Chytriomycesh:0.
009188):0.093285,Obelidium:0.116305):0.330428,(GorgonomycesA:0.333568,GorgonomycesB:0.707554
):0.107599):0.138292,Globomyces:0.490704):0.132253):0.124842):0.227266,((Choanoeca:0.174211,
Monosiga:0.236119):0.128606,Mylnosiga:0.465764):0.123734);
end;
```

Figure S5. ML tree of long TPPPs

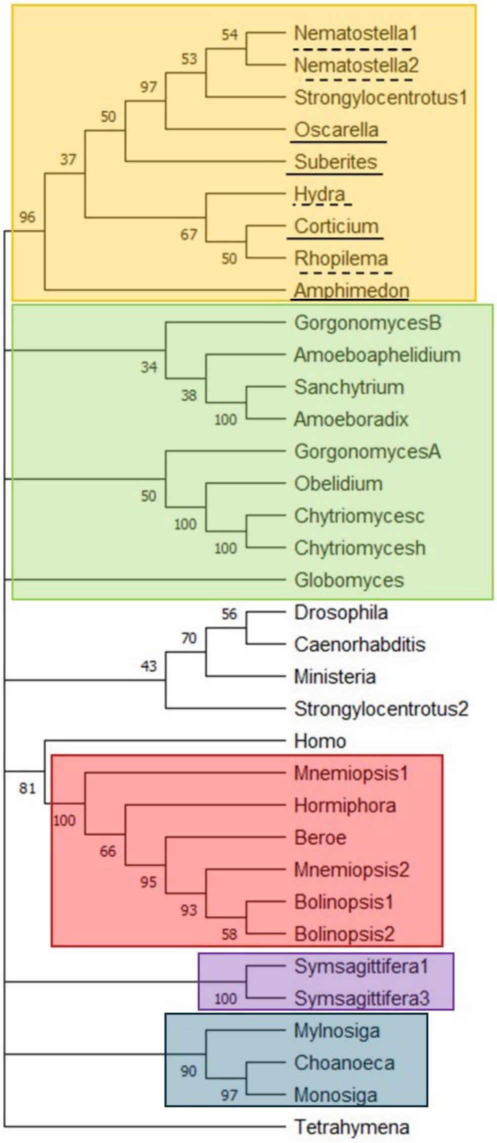

Tetrahymena thermophila short TPPP was used as out-group. The Accession Numbers of proteins/TSAs are listed in Table S1. Bootstrap values (1000 replicates) above 33% are shown at branch nodes. Branches corresponding to partitions reproduced in less than 33% bootstrap replicates are collapsed. Color code is the same as in Fig. 3.

```
(((((((Nematostella1,Nematostella2)0.5400,Strongylocentrotus1)0.5320,Oscarella)0.9700,Suberites)0.5080,(Hydra,(Corticium,Rhopilema)0.5020)0.6720)0.3760,Amphimedon)0.9660,(GorgonomycesB,(Amoeboaphelidium,(Sanchytrium,Amoeboradix)1.0000)0.3820)0.3420,(GorgonomycesA,(Obelidium,(Chytriomycesc,Chytriomycesh)1.0000)1.0000)0.5020,Globomyces,(((Drosophila,Caenorhabditis)0.5620,Ministeria)0.7000,Strongylocentrotus2)0.4380,(Homo,(Mnemiopsis1,(Hormiphora,(Beroe,(Mnemiopsis2,(Bolinopsis1,Bolinopsis2)0.5800)0.9300)0.9580)0.6620)1.0000)0.8180,(Symsagittifera1,Symsagittifera3)1.0000,(Mylnosiga,(Choanoeca,Monosiga)0.9720)0.9080,Tetrahymena);
```

Figure S6. Multiple alignment of fungal and 'double' type TPPPs

```
#NEXUS
[TITLE: Written by EMBOSS 11/08/25]

begin data;
dimensions ntax=36 nchar=510;
format interleave datatype=protein missing=X gap=-;

matrix
Lamellibrachia       ikaafklfatknnga-----------------eatskditrwctdag---
Acrasis              lqqvyvdfacfgkgv-------------ngttgmdgkafskfckdck---
Stylonychia          lkhvfdsyng-------------------taktmegknfvklakdck---
Halteria             lnsvfeayai-------------------drihlegkqfakifrdcs---
Physarum             LRHIYQEFASFGATRGS------------AMPMMDGPKFAKFAKDSK---
Geodia               ledafreycsfgdkd-------------slpltmdgakfakmcrdck---
Dysidea              lerlfvrfcnfgtgn--k----------dmpalmdgtkfakfckdmg---
Halichondria         lesvfknfcsfgagk-------------dgkaqmdnakfaklfrdlk---
Ephydatia1           ledifkkfcsfgakd--------------spssmdgtkfakfckdmk---
Alveopora1           IEQVFNSFCAFGAGS--R----------GAAAEMDGAKFGKLFRDLK---
Paraphelidium_tribon LQDSFEQFAQFGSSKNN-L---------SQGTTMDNAHFAKMCRDAK---
Salpingoecak         MEELFSRFaafgaga--a----------atagELDGAKFAKLCRDTK---
Salpingoecap         MEDAFVNFASFGSGG--K----------KL-HDITLTNYLKMLKATG---
Diaphanoeca          -MAATIIFSQFTGDAHGG----------AKKAQMDGRQFSKMAKDCVIKD
Stephanoeca          LVEMFCLFAG-----VQG----------TETGLMDGRQFVKLLRDTN---
Savillea             LLDVFCSFCG-----AD-----------AEKGQMDGRQFAKMTRDCK---
Helgoeca             LLDVFCAFCG-----VD-----------AEKGQMDGRQFAKMTRDTN---
Acanthoeca           LLEVFCLFCG-----VD-----------AEKGQMDGRQFAKLMRDTG---
Tunicaraptor         MKQLFEAFVSFGA-GSNG----------PAAMEMDSAKLTKFCKENS---
Batrachochytriumd3   lymtfgsfcqfgssrnl-sg---smtdi-sgptmdgskwakfcrdtg---
Spizellomyces1       lreiyeafcafgsnrnl-snphgsmesi-agptmdgakfakfardnr---
Powellomyces         lreiyeafcafgsnrnl-ats----tdv-sgptmdgakfakfardnk---
Chytriomyces2        lharfekfcafgrgsvg-ssld-sl--t-sgstmdgakwakfardcn---
Chytriomyces3        saaafeefssfgasrhpigspgpvphha-qgiamdsskfaklcrdsg---
Rhizoclosmatium1     lraafeefalfgtpktiaprpahl--pn-ppiqmdskcfaklcrdsg---
Synchytriumm         lrtvydsfcafgssrnl-ssnsmsmesl-tgaqldnakfaklskdtg---
Gervar1417039        LKEVYVAFCAFGSNRNL-ASS----SDV-QGPTMDGAKFAKFARDNK---
Gaesem1531638        LREIYEAFCAFGSNRNL-SS-TGSLENI-AGPQMDGAKFAKFARDNK---
Obemuc1859513        LRTAFEEFALFGSPRTQAHS-----TTT-APVQMDNAKFAKLCRDSG---
Obemuc1832726        LRNTFERFCAFGRGSVG-SSLD-SL--T-GGSTMDGAKWAKFARDSN---
Glopol1609812        LYGVYESFCQFGSTRTSNASLSSSLNNL-KGPTMDGSKWAKFCRDCK---
Clapol1_1869731      LRSAFEAFASFGSSRNLAASAP-IP-NA-AATTIDSARFAKLARDTG---
Clapol1_1821589      LHDVFERFCSFGSNRNL-ASMDASF--G-PGLTMDGAKFAKFARDSG---
Catenaria2           lrelfeqfasfgtpranasgpn-----fqqetvmdsarflkffrdqn---
Parsed11082034       LYEVFTQFCAFGASRNSNPNLSGSLNNLSSGPQIDNVRFSKFFRDLK---
Olpidium             ----fqsfcafgssrnl-san----txx-xxphqdnqkfakfaretk---

Lamellibrachia       ------vfg-ktcnsnnldiafsk--vkp---kgkntitakeidaliael
Acrasis              ------lin-skfsatdvdlifantkvkp---kaerkisyeqflvaleli
Stylonychia          ------iid-kkltatdvdlifak--ikd---kserkityaqfekgleff
Halteria             ------lid-kkltqpeidiafak--akq--ikterklnfeefllaldfc
Physarum             ------LLDKTNLTATDVDLIFAK--AKS---KTERRISYDQFYTAVTML
Geodia               ------lld-kkltptdvdiiftk--akp---kaerkltleqfeqavrml
Dysidea              ------lvd-krfpatdvdivfaq--skp---kterkinfhqfkvtmeli
Halichondria         ------lld-kkftatdvdiifsktevkp---kaerkinfeqfrkavmla
Ephydatia1           ------lld-knltttdvdiifak--skp---kterkmtleqfevalgfl
Alveopora1           ------VLD-KKLTSTDIDIIFAR--VKA---KTDRKINFDQFCEALKLC
Paraphelidium_tribon ------VLG-KSVTTVDVDLVFKK--VLI---KGERRINFDQFKAALEML
Salpingoecak         ------IIS-KKTSSTDVDIIFSK--VKP---KTARKINYEQFLQALDLI
Salpingoecap         ------IID-KHYPTVDAELLFAK--GGS------KTMSFDKFRQalati
Diaphanoeca          GETAWSLLD-KKFTSTDIDIIFSK--VKA---KGERKINVVEFTNALTEI
Stephanoeca          ------LVT-KKFTATDVDIIFAK--AKE---KGMRKITYDQFCDALRMV
Savillea             ------MLD-DSFTSIDTDIIFAK--CKA---KAMRKIDFHQFLDAINMC
Helgoeca             ------LID-KKFTSIDTDIIFAK--VKA---KSLRKIDFHQFLSAIEMC
Acanthoeca           ------MLT-KKFTSIDTDIIFAK--VKA---KALRKIDFNQFLAAAELC
Tunicaraptor         ------VFN-KKCTSTDSDIIFSK--VKT---KGARKIVFSEFVAALQLL
```

```
Batrachochytriumd3   ------iid-khitttdidiwfnk--vka---ktvrkidfeqfqaalhlv
Spizellomyces1       ------lidnkrvtttdvdiifnk--vkt---kgarkldwntfidgltei
Powellomyces         ------lidnkkvtstdvdiifnk--vkp---kgarkldwntflegltqi
Chytriomyces2        ------lidnkrvtstdidiifnk--vka---ksarridweefqvavklv
Chytriomyces3        ------lidnetftlidcdivfsk--ckk---vgkrkleyddfrralaei
Rhizoclosmatium1     ------liddislslidvdiifak--vkq---pgkrklgyadfqralrei
Synchytriumm         ------vvdgkkitnadiditfnk--vka---kgarkldwesfqdalall
Gervar1417039       ------LIDNKKVTSTDVDIIFNK--VKP---KGARKLDWNTFLEGLTQI
Gaesem1531638        ------IIDGKKVTTTDVDITFNK--VKP---KGARKLDWNTFLDALTQL
Obemuc1859513         ------LVDNISLSLIDVDIIFAK--CKQ---VGKRKLFYADFQRALREI
Obemuc1832726        ------LIDNKKITSTEIDIIFNK--VKA---KNSRRIDWEEFQAALKMV
Glopol1609812        ------VID-KVITPTEVDIVFNK--VKS---KTERKIDYDQFIEGLKMI
Clapol1_1869731      ------LIAPPSLTIVDVDLIFEK--CKI--AKGTRKLDFKGFKKALQMV
Clapol1_1821589      ------LIDGRKVTTTEIDIIFNK--VKA---KTARRIDWNEFVKAVQML
Catenaria2           ------lld-tnltptsldlhftk--akasfkrtdrklpwnaflyalqla
Parsed11082034       ------VLN-KELTPTDIDIIFNK--VKK--TKTDRKLDFDCFKNALVLC
Olpidium             ------lisapkvtavdvdnafMR--VKP---RASRKIDFEGFQQALRIL

Lamellibrachia       -akkykddkkcdeaeavskinek-ms--navpkghgt---tktsktgnvg
Acrasis              aakkg---------ctmdsltss-iqs-tngpstnnt---qkvdesgivs
Stylonychia          aekkg---------ctpadivek-ila-tggpqfsgt-------qadhvk
Halteria             aqkkg---------iptetlayelvar-cqgplyvat-------itdqvr
Physarum             ADKRYPD-QT--GEGAFEAIMKKIITS-QGPSASSAT----VADSSGIYG
Geodia               sekkypgdp-----kamqklqsk-ltt-ptt--hpga---tkvagnavld
Dysidea              atkkygddp-----ngltklhqt-lkl-fkgpqnsat---vqpsdspvvv
Halichondria         aeikypgda-----dcvkklegk-iis-gagpgssspsakvsaasgsvvd
Ephydatia1           aekkypgqp-----dgveklkar-iat-gsgplthgv---tvtdtsegtq
Alveopora1           AEVKYKGDA-----DGVKKLKKK-ILD-GSGPRTAHP---VKTSNKDTVD
Paraphelidium_tribon AAQKYPDDAS--ASEKVFSL-----IA-AATPAQNGT----QADASGIYS
Salpingoecak         AAQKG---------MTVERLHEK-MLE-NAAPTNSGT----ASANSSIVD
Salpingoecap         aaakk---------ttveaLEEA-LVa-sggpsasgt----sptksaVVD
Diaphanoeca          SVKKN---------MTFAELCER-IME-SGGQKFTGT-------KATHVK
Stephanoeca          AEKKK---------MTFESLVLK-LVT-AEGPVLKGT-------SAQKVA
Savillea             AATKR---------VSPSALAAK-MVK-AGGPKIGGT-------HAGKVR
Helgoeca             ATSKR---------VPVSDVVAA-LVT-GGGPKMTGK----SPARSTKVR
Acanthoeca           AGAKK---------VPVSDVVAT-LTS-GGGPKVAGT-------RTDKVR
Tunicaraptor         AEKRG---------SAYEDVVDK-AIE-KGGPASSGT-------KAQPNK
Batrachochytriumd3   aakrygsskp--pteaynllvrsilns-garpvatgt----ittsdsvtq
Spizellomyces1       avkkypg-kq--grqaldavistimkk-gggpiatgt----tpksdgivd
Powellomyces         aekknsg-kq--grealdsligliikk-gggpiatgt----apksdaivd
Chytriomyces2        agkkypk-mh--eqdaytktiydacil-sngpvarat----tvkndpvld
Chytriomyces3        adirqch---------vshvva--mvv-saapeingt----lphadelid
Rhizoclosmatium1     awtrqcd---------eehvva--lvv-sapvlingt----ipqvdeite
Synchytriumm         aekkyat-lp--aeqafsrlvtdilsn-gsphltnta----vpknsaivd
Gervar1417039        AEKKNPG-KQ--GRDALDSLIGLIIKK-GGGPIATGT----APKSDAIVD
Gaesem1531638        AEKKYAG-KK--GREALEAVTGDIVKR-GSGPIASGT-VRDTPQSDAIVD
Obemuc1859513        ADTRQCD---------IDHVVA--MVV-TAAPELNGT----IPQVTELIE
Obemuc1832726        AEKKYAE-KH--AQDAFTQVMYDVCVR-AHGPVSKAT----AVQKDAVLD
Glopol1609812        SAKKYSN-KT--TTESFVILVQFVLST-RGAPVVQTN-LKNSVQ-MDVTN
Clapol1_1869731      AEKRQCD---------YSHVVA--IVV-AGSPALTGT----IPDYDPVIE
Clapol1_1821589      AEKRYPN-KR--PQDALDSTLYDVCIK-AKGPVSSGT----AVKNDAVLD
Catenaria2           attkygsams--dadaleqlineavks--qgpiarat----vpvadgvyg
Parsed11082034       SEKKYRPKMD--GYQAYEKIVTDIVSKDNAGPLLHGT----RPEASGVYS
Olpidium             AEKRYPQ-KD--PEEAYLTLVNHVCQS-KG-PKVHSI----QPEATGIFE

Lamellibrachia       kmtdtsqytgshkerfda-dgkgkgiegrenladnsgyvgnykgs-gtg-
Acrasis              kmtdaslytgahksrfde-egkgkglegrdsipkgtgspsspi-spks--
Stylonychia          fhddkslytgvyaqg-------------gptnv-------------dqin
Halteria             lhddkttytgvyaqg-------------gprtv-------------dgsp
Physarum             KLTDTNLYTGAHKERFNA-DGTGKGLDGRDKVVKGTG-------------
Geodia               rmtdtskytgshklrfne-sgkgkglegrdsvakgpghipalv-saqt--
Dysidea              rvtdtakytgshklrfde-egkgkglagrdslvkgkgqlps-v-aaqe--
Halichondria         rltdtskytgshkerfda-sgkgkgiegradpaagsg----------n--
Ephydatia1           rmtdtskytgthklrfde-egkgrglagrdsvakghgmspgsv-ssqq--
Alveopora1           RLTDTAHYTGSHKLRFDE-EGKGKGLAGRDSVPKGAGRSPGCV-AGQE--
Paraphelidium_tribon KLTDTSLYTGSHKLRFDA-DGKGRGIEGRDQP--VQ--------------
Salpingoecak         KLTDSSLYTGAHKERFDA-DGKGRGLAGRDSTAKGAGHVG--GVHPGGGG
```

```
Salpingoecap         RLTDTSQYTGAHKERFDA-DGKGKGLAGRESVSKGAGALPNKSLNPGPGH
Diaphanoeca          LHDDKAGYTGVSSRG-------------GPSTV-------------DELN
Stephanoeca          LHDDKSLYTGVYANG-------------GPSTV-------------DSDR
Savillea             LHDDKAGYTGVAANG-------------GPVTV-------------DTGR
Helgoeca             LHDDKAGYTGVHARG-------------GPSTV-------------DGER
Acanthoeca           LHDDRGGYTGVHSKG-------------GPSTV-------------DSER
Tunicaraptor         FYDDKSNWTGTAAKG-------------GPSTV-------------DLAN
Batrachochytriumd3   rltdhthytgthknrfde-agqglglagrdth--sr--------------
Spizellomyces1       rltdtskytgthklrfde-agrgrgaegrdrp--sa--------------
Powellomyces         rltdtskytgthklrfde-agqgrgaagrdrp--sa--------------
Chytriomyces2        rltdvsgytgthknrfds-agrglglngrvtg--ks--------------
Chytriomyces3        kltdtslytgmhrarfdpvtgqgrglagreti--sp--------------
Rhizoclosmatium1     kltdvslytgthkerfdpvtgqgrglagrdti--nt--------------
Synchytriumm         rltdtstfpvthknrfed-kglgsqga--lna--kss-------------
Gervar1417039        RLTDTSKYTGTHKLRFDE-AGQGRGAAGRDRP--SP--------------
Gaesem1531638        RLTDTSKYTGTHKLRFDE-QGHGRGLAGRDQP--AK--------------
Obemuc1859513        KLTDTSLYTGTHKARFDPVTGQGLGLDGREPV--NP--------------
Obemuc1832726        RLTDVNGYTGSHKNRFDS-AGRGLGLQGRDTI--SR--------------
Glopol1609812        RLTDPQKYTGIHKEKSH-------GTMGSVYDLSDSG-------------
Clapol1_1869731      KLTDTSLYTGTHKNRFDPETGVGRGLEGREPV--NT--------------
Clapol1_1821589      RLTDTHAYTGTHKLRFDA-DGHGRGMEGRDQP--SK--------------
Catenaria2           kltdasqytgqhkdkpsg-qsegkgytiesplaksra-------------
Parsed11082034       KLTDHTLYTGSHKERFDE-EGRGKGVSGRNDS--SIG-------------
Olpidium             kltdtklyTGAHKARFDD-NGVGRGAAGQVDA--EDA-------------

Lamellibrachia       ypli-------tswvk-nvps-----------------------------
Acrasis              --------------------------------------------------
Stylonychia          ptvt---fgsaphn--------------------------------de--
Halteria             k-vt---isklgle--------------------------------ef--
Physarum             SNRGVAPVNLQFRAs-----ddpsppvsddEETNE--------------A
Geodia               ------------syvs-gnligldalds----------------------
Dysidea              ------------pyvq-gfksssspkl-----------------------
Halichondria         ------------gyvs-gykaggsygkv----------------------
Ephydatia1           ------------ayvs-gykhegtysld----------------------
Alveopora1           ------------GYVS-GYKGEGTYDKS----------------------
Paraphelidium_tribon TSDLSQIVNRKSGTAA-ATTGGKRSPTASMEKING--------------S
Salpingoecak         VKDLSELLDRSTADVR-GVKHDQHDDGKGTMRAAA----PAAAA--TTSA
Salpingoecap         VRDLSELTDRTPSDVR-GVKYAEGESP-----------------------
Diaphanoeca          VADLSVFTERTPHGP-----------------------------GKGL--
Stephanoeca          TVDFGKQMGRDTADVR-GVVIGGFDTTPHLSKT-----HAAADGGEGV--
Savillea             APSMGKPSTPREHAHH--VANV----TATL--------------------
Helgoeca             HIDFGEPSPRAPSAKV-STVTA------SL--------------------
Acanthoeca           QIDFGTPPPRAAKASS-GVASA----TSQM--------------------
Tunicaraptor         Q-DLSKHLDRTDADVR-GVKKAATTTARPTSAGGSSARPPSAKGARPVSA
Batrachochytriumd3   tnelskivnrkeadir-gvplaaspigsprgsv-----------------
Spizellomyces1       tdqlskitnreetsvr-glpvsi----dpeegdk----------------
Powellomyces         tsdlskitnreetslr-glpvsi----dpnek------------------
Chytriomyces2        tdtlskivnrdapsqq-plaqtnrqsvsnrrqesq--------------s
Chytriomyces3        tadlsaivsrhnsattlgskas----------------------------
Rhizoclosmatium1     tanlstivsrhntartlgskgp----------------------------
Synchytriumm         tgt--tptkspgggvk-gspvg----------------------------
Gervar1417039        TSDLSKITNREETSLR-GLPVSI----DPNEK------------------
Gaesem1531638        TDDLSKLVNREPTTVR-GLPVSV----DPDAQEQ----------------
Obemuc1859513        TANLSSIVSRHNTARTLGSKAL----------------------------
Obemuc1832726        TDTLGKIVSRDIAVQR-PSVPQNSRPITTTE-------------------
Glopol1609812        RESISTIKTTNG--------------------------------------
Clapol1_1869731      TSNLANLVSRHNPNKTLGAPTP----------------------------
Clapol1_1821589      TDRLDKLVNRDGAG------------------------------------
Catenaria2           tgs------rsnstsn----------------------------------
Parsed11082034       VKRLDKLVSRDPAVQNRAASMN---------Q------------------
Olpidium             TRNLSTIVS-----------------------------------------

Lamellibrachia       -------------------npkllcr-----lssrdihvtqtsemgdlat
Acrasis              --------------------psk---------------------------
Stylonychia          ----eeekvptkr--------maq-------------msvnnnpagslke
Halteria             ----kn--dydks--------pkv-------------sfvaaqpastlee
Physarum             ----------------------LAE---QVAQLGVsksksaspakpalsk
```

```
Geodia               ------e--------rakerpraka----------------kaaaearas
Dysidea              -------------------spytsk----------------kassspaas
Halichondria         ------n--------khcgekgksr-------------sttthwnmsles
Ephydatia1           -----------------ksspktea----------------kvkkmptkt
Alveopora1           -----------------PKQPS----------------------------
Paraphelidium_tribon -KAA----------------NTATS---------NKNLSGKSTGNLVKS-
Salpingoecak         RTSSASAP----SGGSPKKRPTTSGSASSGGPSGGTVVKGSSDGYDSLEE
Salpingoecap         -----------------PQRKSTSS----------T--VISSPEAKSLDE
Diaphanoeca          ----QGSPSPKKS--------PKQH-------RN-VETISTSAAAESLEQ
Stephanoeca          ----THAASPTKNSPRTVQAPPPTA-------VA-RPSSAETATSGTLEG
Savillea             -----------------------------------HA-THVVEGEYTLQT
Helgoeca             -----------------------------------QETHITEGGEYTLKM
Acanthoeca           -----------------------------------QS-MQVSDGGYTLKM
Tunicaraptor         TKDSVKSPRPGSRPGSAVKRPGSSA-------SSKNTRLDPETTEGQLAE
Batrachochytriumd3   --------------------tmsys---pkskrahaavttmstealdaea
Spizellomyces1       --------------------qelts---sagkrghssvvtasserldlas
Powellomyces         ------------------------g---spakrqhsnvvtasserldnaa
Chytriomyces2        tmra----------------ptaas---sisqnkrasvltqseekleniv
Chytriomyces3        ---------------------------------------------aanra
Rhizoclosmatium1     ---------------------------------------------lspss
Synchytriumm         ----------------------------------nkrsvtesmdklelaa
Gervar1417039        ------------------------S---SPAKRGHSSVVTASSERLDQAA
Gaesem1531638        ---------------------SKPS---SGSKRGHASVVTASSEKLDVNA
Obemuc1859513        ---------------------------------------------PTPHA
Obemuc1832726        --------------------KNRTS---TVASGKRQSVLTQSEERLENIT
Glopol1609812        RRYS----------------NFGQE---TSTKRGFNNVVTASTEYLDMNS
Clapol1_1869731      ---------------------------------------------PKSPA
Clapol1_1821589      ----------------------------TAASTKRQQVLTQSEEQLDAIA
Catenaria2           ---------------------llda---stgtrg-------stgalnkst
Parsed11082034       -----------------------------TNKR----GISASMEAMDAQG
Olpidium             --------------------------------------------------

Lamellibrachia       tfksfatkangae-------attkditrwftdcgiltkk---scnsnnld
Acrasis              --------------------------------------------------
Stylonychia          vfegf---t------ggaaemdgktfakmskdtkildka---lta-tdid
Halteria             vyagf---t------fsqpdmdgktfvklfkdckildkk---ltt-tdld
Physarum             s------------------------------------as--s-----GKT
Geodia               pktqks--ta---------------------------tk---ssp-qksp
Dysidea              ---kkp--sssps--a----------------------------------
Halichondria         vfksfcsfgagkd--g-kaqmdnakfaklfrdlklldkk---lta-tdvd
Ephydatia1           eksdrp--sdgkd--k----a--------------qdkt---dsp-ekvq
Alveopora1           --------------------------------------------------
Paraphelidium_tribon N------------------------------------KN--L-----SAS
Salpingoecak         LFQSFASFGVGSS--AKVKEIDGAKFVKLCKDCKLVDKK---TTT-TDVD
Salpingoecap         LFHRFCSISGGSS----NKEMDGSKFAKLCRDCGLIGKK---LTA-TDVD
Diaphanoeca          VFLGF---T------NGAKEMDGRQLAKLTKDAKILNKK---ITA-TDVD
Stephanoeca          VFSEH---S------LGAKEMDGRQFVKLCKDSNLLSKKK--FTS-TDVD
Savillea             FFKDH---S------GGAKEMDGRQFAKMARDTKLIDKK---FTS-IDLD
Helgoeca             FFTDH---A------GGGKDMDGRQFAKMAKDTKLIDKKPPGLTT-IDLD
Acanthoeca           FFTDH---S------LGAKEMDGRQFVKMAKDTKIVDKKPPGLTT-TDLD
Tunicaraptor         LFTTFCAFGGGVQPSYLSAEMDGAKFVKFCKDSKLVGKK---MTV-TDVD
Batrachochytriumd3   n------------------------------------rp--k-----kav
Spizellomyces1       s------------------------------------kp--k-----ssk
Powellomyces         s------------------------------------qp--k-----ssk
Chytriomyces2        n------------------------------------ap--k-----kap
Chytriomyces3        l------------------------------------sp--p--------
Rhizoclosmatium1     s------------------------------------fs--a-----sap
Synchytriumm         n------------------------------------pp--k-----snk
Gervar1417039        S------------------------------------KP--K-----AGR
Gaesem1531638        H------------------------------------KP--K-----ATK
Obemuc1859513        S------------------------------------HP--S-----SPT
Obemuc1832726        N------------------------------------AP--K-----KTP
Glopol1609812        N------------------------------------KT--K-----KQE
Clapol1_1869731      M------------------------------------SR--T-----AST
Clapol1_1821589      Q------------------------------------AP--K-----KA-
Catenaria2           s--sp---r------gsksn---------------lyap--tts--snak
Parsed11082034       P-------T------KSIKN---------------LELP--N-----DAS
```

```
Olpidium             --------------------------------------------------

Lamellibrachia       icfs-----kvk-qkgkntitskemtal-iaevakayktdhklgsdgeav
Acrasis              --------------------------------------------------
Stylonychia          lifa-----kvk-dkaarkinyaqfqkg-ieecatkkkit---------f
Halteria             ilfs-----kik-akgqrkigfeqflta-lqeisqlksit---------f
Physarum             PAKSPAAS------------------------------------------
Geodia               ava------kss-prvv--------skt-gasspaakpst----------
Dysidea              --------------------------------------------------
Halichondria         iifs---ktevk-pkaerkinfeqfrka-iklaaekkypg-----dadcv
Ephydatia1           agkscgekahra-peg---------kat-geklhedkppg-----ek--s
Alveopora1           --------------------------------------------------
Paraphelidium_tribon PAGQVQ--------------------------------------------
Salpingoecak         IIFA-----RCK-PKGARKANYEQFVHA-LELIAEQRKTT-----V----
Salpingoecap         LIFT-----KCK-DKSARKIDMQHFNKA-IHLIAEHMHLP-----F----
Diaphanoeca          ISFA-----KFK-SKGARKINYTQFEQV-IESFALKEKIT---------K
Stephanoeca          IIFA-----KAK-GKGSRKLTFPQFEHA-IAEIATALRVD---------A
Savillea             IIFA-----KVK-AKASRKITFKQFKDA-ITLCAEKKGKS---------F
Helgoeca             IIFA-----KVK-DKSARKITFKQFREA-LKACAEKKHMY---------Q
Acanthoeca           IIFA-----KVK-AKSARKINFAQFQEA-LVHCAEKKHVY---------L
Tunicaraptor         LIFT-----KSKFDKTDRKITWDSFRQAALPQMAEKLGTS---------V
Batrachochytriumd3   tsqaprtvk-----------------------------------------
Spizellomyces1       lgsntnlankpkt-------------------------------------
Powellomyces         lgsnsnlag-----------------------------------------
Chytriomyces2        iagrrsqaphattkggny-------------------tts----------
Chytriomyces3        --------------------------------------------------
Rhizoclosmatium1     iia-----------------------------------------------
Synchytriumm         algsmdnms-----------------------------------------
Gervar1417039        IGGSNSNLNKGSS-------------------------------------
Gaesem1531638        PAEKTKKAE-----------------------------------------
Obemuc1859513        --------------------------------------------------
Obemuc1832726        VAGRRAT-----QQSANY-------------------TTS----------
Glopol1609812        MYSSNTQLPKVNF-------------------------------------
Clapol1_1869731      PNR-----------------------------------------------
Clapol1_1821589      IAGRRAANPVPSSKFGS----------------------S----------
Catenaria2           iagskgslagr-t-kgaa-------------------gtg----------
Parsed11082034       LSHSRSNSGA--SSNGLN-------------------KSN----------
Olpidium             --------------------------------------------------

Lamellibrachia       aqmte-klaggkpm--------ahgttkasatggvdrmtdtskytgshke
Acrasis              ------------pst--plsnpptvtktqtssgifarltdtskytgahkq
Stylonychia          eqleekilavggpvf---------tgtktd---kvkfhddkslytgvyaq
Halteria             dqlknqicsvggpvf---------tgtrae---kvkwhddkstytgvyak
Physarum             -P----MTKTnsans--gslsksaskpaaskpsIFDKLTDSSQYTGSHKN
Geodia               -ktk-------tsav--atsspksspaskpkgdiydrltdtskytgahkh
Dysidea              -------------sk--kpasspstakkptsspvvsrltdaskytgthkl
Halichondria         kklegkiisgeg-----pgssspsarvrtasggvvdrltdttkytgshke
Ephydatia1           gkavgtkvsgeeske--skasvgksqaqgekkdiydrltdpstyhathkh
Alveopora1           ---------------------------RPKSGDIVDRLTDTSKYTGSHKQ
Paraphelidium_tribon --------K-SNKNL--SVENSLSAAPKSKSGDVYDRLTNTSGYTGSHKE
Salpingoecak         EKLHAKLLKAEGPRL---------SGTIADQGGVLDKLTDSSLYTGAHKH
Salpingoecap         DEVEQMMLHAGGPKL---------TGTAADNAHIVDKLTDTHGYTGAHKE
Diaphanoeca          EVLVERILAKGGPTF---------SGTKTQ---GSRLHDDKSTYTATQAH
Stephanoeca          SEVVARLVANGGPVF---------SGVRTD---AVRLHDDTDGYTGVYKQ
Savillea             EELEDYLLKSGGPVF---------TGVKTD---KVKLHDDKSTYTGVYTH
Helgoeca             DALEEHLVASGGPVF---------TGVKTE---KVRLHDDKAAYTGVYSH
Acanthoeca           EDLEAHLVAFGGPVF---------TGVKAD---NVKLHDDKSTYTGVYTH
Tunicaraptor         EQVMEKAVAAGGPTS---------SGTKAQ---SNRFHDDKSLYTGTHAK
Batrachochytriumd3   -p----iv--ksset--llntttkktvgassgnvfdrltntkgytgthke
Spizellomyces1       ------------pqa--kidksyga--nakggsvfdrltnsgqytgthkh
Powellomyces         --------k-pkpps--asaqsygtnnpkaggsvfdrltdssgytgahkq
Chytriomyces2        ss----l-a-ssqqs----laks--sttaskssvfdrltdssgytgthqh
Chytriomyces3        -------------------phertaasssagpsvfdrlnnvksftgthkh
Rhizoclosmatium1     -----------------wgenrsmsgsedgkpsvfdrlndvstftgthkh
Synchytriumm         -p----l--------------gasksksgsvgsvydrltdtkqytgthkl
Gervar1417039        SN----LSK-QSSSV--EAKQSYGTANAKAGGSVFDRLTDTTGYTGAHKQ
Gaesem1531638        ----------PKPYT--A-VTKTSCGQSSKGGSVFDRLTDSSGYTGSHKE
```

```
Obemuc1859513        -------------------SPVRYSEDQGGRESVFDRLNNVSTFTGTHKH
Obemuc1832726        SS----L-A-SSQQS----LATKNSSVSSSKASVFDRLTNTKDYTGAHKH
Glopol1609812        -------------GS--KQNLTSSNSNLNQNGNVFDRLTNTQGYTGTHKQ
Clapol1_1869731      ------------SGS--EGSLGSAPSSPSARPSVFDRLTSVSTFTGSHKH
Clapol1_1821589      TS----IKS-STPGS----ITTLNKSVTGSRTAVFDRLTDTSGYTGSHKH
Catenaria2           sh----t----tl----gsseyvptqpsipkgsvydrltnpkgytathke
Parsed11082034       SN----LTRSKSSGLSGSSSQLKTTVPQVPAGSVFDRLTDVKKYHGTHKH
Olpidium             ---------------------------RDPAKNVFDRLTDPKGYRGTHAQ

Lamellibrachia       rfdaegkgkgmagredlhdn------------sgyvgaykdayssrrrrf
Acrasis              rfdedgkgrgaagrdiggng------------tgtqggrvgdlssmtrty
Stylonychia          ggpst-ida---gngmis---------------disq-lcdrsdanvrgv
Halteria             ggpet-vdi---grtmin---------------dirh-lcdrseadirgv
Physarum             RFNSDGTGRGMAGRDSIRKG-G-------VSGQDLSQMVRRS--------
Geodia               rfnetgrgkgldgrdapakg------------aamtpspasnltsyvagy
Dysidea              rfdergkgkglagrdapskg------------ga-aqvpvssqaayvqgy
Halichondria         rfdgtgkgkglegrdssakg------------rgmvagsvadqaayvsgy
Ephydatia1           rfdssgkgrglegrdapakg------------agmspgsvksqaayvtgy
Alveopora1           RFDGSGKGRGMAGRDLPAKG------------SGMSPGSVSKQAAYVTGY
Paraphelidium_tribon RFDANGNGKGLAGRT-----------------------------------
Salpingoecak         RFNEDGKGRGIAGRTANEGV------------KDMSHVL-DRSEADVRGV
Salpingoecap         RFDADGKGKGLEGRVQATGT------------ADLSDLT-DRTAADVRGV
Diaphanoeca          GGPTL-VGRGFRGEGEVD---------------DLSQ-LADRTSADVRGT
Stephanoeca          GGPAV-TGD---HGPRVD---------------SIAT-LCDRSPADVRGV
Savillea             GGPDT-KSL---I--AHH---------------DITN-LTDRTQADVRGV
Helgoeca             GGPDT-GSL---T--AHH---------------DIST-LTDRSQADVRGV
Acanthoeca           GGPDT-GSM---G--AHH---------------DIST-LTDRTPADVRGV
Tunicaraptor         GGPST-VDH------ANQ---------------DLSA-HLDRAPADVRGV
Batrachochytriumd3   rfhgss--nsihg-------------------------------------
Spizellomyces1       rfnadgsgrgiagrdspakg-tspgsyrggdvkdlsqilrs---------
Powellomyces         rfnadgtgkglagrdapakg-nspgkyrggdvkdlsqilrn---------
Chytriomyces2        rfnadgsgrglagrdsaplgkggviqyrggnvnslsqilrs---------
Chytriomyces3        rfnsdgtgrgkagrd-----------------------------------
Rhizoclosmatium1     rfnpdgtgrgkegrdspsst---------ad---lsqivsark-------
Synchytriumm         rfnddgtgrgiagrdspskg-gvgpvrdgqnvndlsqilrr---------
Gervar1417039        RFNADGTGRGIAGRDAPAKG-GSPGKYRGGDVKDLSQILRN---------
Gaesem1531638        RFNSDGTGRGLSGRDSPSKS-GAAGKYRGGDVKDLSQILRS---------
Obemuc1859513        RFNEDGTGRGKAGRED----------------------------------
Obemuc1832726        RFNADGTGRGLAGRDSAPLGSGGESKYRGGDVKDLKQILRT---------
Glopol1609812        RFDEHGNGRGLEGREAIAKGSGTQSIYRGGNVNSLSQILRS---------
Clapol1_1869731      RFNADGTGRGKEGRVGDGAG---------DVVSDLSQITRR---------
Clapol1_1821589      RFNADGTGRGAAGREIVSKGH-SVGTYRGGDVKDLSQILRN---------
Catenaria2           rfddqgkgkgkagrvqddlgt-----------kslehlvark--------
Parsed11082034       RFNEDGSGKGKAGRVQDVGP------------KKLESFLRN---------
Olpidium             RFDEQGKGRGLAGRDSTSKG-AGPGAYHGGDVKDLSQILRS---------

Lamellibrachia       -deaecsrk-
Acrasis              lnk-------
Stylonychia          kkh-------
Halteria             kkh-------
Physarum             ----------
Geodia               khentygk--
Dysidea              rnegtyggkk
Halichondria         kaegsygkk-
Ephydatia1           khegtygkak
Alveopora1           KNEGTYKKQ-
Paraphelidium_tribon ----------
Salpingoecak         VPRAE-----
Salpingoecap         KKH-------
Diaphanoeca          KH--------
Stephanoeca          KQ--------
Savillea             KRH-------
Helgoeca             KR--------
Acanthoeca           KR--------
Tunicaraptor         KK--------
Batrachochytriumd3   ----------
Spizellomyces1       ----------
```

```
Powellomyces          ----------
Chytriomyces2         ----------
Chytriomyces3         ----------
Rhizoclosmatium1      ----------
Synchytriumm          ----------
Gervar1417039         ----------
Gaesem1531638         ----------
Obemuc1859513         ----------
Obemuc1832726         ----------
Glopol1609812         ----------
Clapol1_1869731       ----------
Clapol1_1821589       ----------
Catenaria2            ----------
Parsed11082034        ----------
Olpidium              ----------
;

end;
begin assumptions;
options deftype=unord;
end;
```

Figure S7: Bayesian tree for Fig. 4

```
#NEXUS

[ID: 9679705640]
begin trees;
   [Note: This tree contains information on the topology,
          branch lengths (if present), and the probability
          of the partition indicated by the branch.]
   tree con_50_majrule =
(Lamellibrachia:0.899154,Acrasis:0.449334,((((((Stylonychia:0.295196,Halteria:0.508894)1.00:
0.136038,Diaphanoeca:0.433846,(Stephanoeca:0.405920,((Savillea:0.264568,Helgoeca:0.143635)0.
73:0.066229,Acanthoeca:0.153811)1.00:0.264249)1.00:0.184616)1.00:0.159376,Tunicaraptor:0.507
644)1.00:0.244331,(Salpingoecak:0.333684,Salpingoecap:0.493830)0.89:0.074065)1.00:0.147003,(
((Physarum:0.498818,((Chytriomyces3:0.248951,(Rhizoclosmatium:0.249042,Obelidium1:0.130378)1
.00:0.112641)1.00:0.169595,Zopfochytrium1:0.289387)1.00:0.346371)0.85:0.115597,Paraphelidium
:0.438564,(Catenaria:0.668074,Paraphysoderma:0.445016)1.00:0.188512)0.74:0.108892,((Batracho
chytriumd:0.390036,Globomyces:0.535402)1.00:0.154468,(((Spizellomyces:0.142452,(Powellomyces
:0.049465,Geranomyces:0.100213)1.00:0.090451)1.00:0.098898,Gaertneriomyces:0.176963)1.00:0.1
63160,Synchytrium:0.550847)0.62:0.083260,(((Chytriomyces2:0.204996,Obelidium2:0.179192)1.00:
0.199007,Zopfochytrium2:0.222816)1.00:0.177848,Chytridiales:0.443834)0.98:0.118100)0.80:0.09
5028,Olpidium:0.457275)1.00:0.276985)0.97:0.117264,((((Geodia:0.449508,Ephydatia1:0.320411)1
.00:0.165201,Dysidea:0.434420)0.99:0.131867,Alveopora:0.222189)0.66:0.142305,Halichondria:0.
280013)1.00:0.155525)0.84:0.137859);

   [Note: This tree contains information only on the topology
          and branch lengths (mean of the posterior probability density).]
   tree con_50_majrule =
(Lamellibrachia:0.899154,Acrasis:0.449334,((((((Stylonychia:0.295196,Halteria:0.508894):0.13
6038,Diaphanoeca:0.433846,(Stephanoeca:0.405920,((Savillea:0.264568,Helgoeca:0.143635):0.066
229,Acanthoeca:0.153811):0.264249):0.184616):0.159376,Tunicaraptor:0.507644):0.244331,(Salpi
ngoecak:0.333684,Salpingoecap:0.493830):0.074065):0.147003,(((Physarum:0.498818,((Chytriomyc
es3:0.248951,(Rhizoclosmatium:0.249042,Obelidium1:0.130378):0.112641):0.169595,Zopfochytrium
1:0.289387):0.346371):0.115597,Paraphelidium:0.438564,(Catenaria:0.668074,Paraphysoderma:0.4
45016):0.188512):0.108892,((Batrachochytriumd:0.390036,Globomyces:0.535402):0.154468,(((Spiz
ellomyces:0.142452,(Powellomyces:0.049465,Geranomyces:0.100213):0.090451):0.098898,Gaertneri
omyces:0.176963):0.163160,Synchytrium:0.550847):0.083260,(((Chytriomyces2:0.204996,Obelidium
2:0.179192):0.199007,Zopfochytrium2:0.222816):0.177848,Chytridiales:0.443834):0.118100):0.09
5028,Olpidium:0.457275):0.276985):0.117264,((((Geodia:0.449508,Ephydatia1:0.320411):0.165201
,Dysidea:0.434420):0.131867,Alveopora:0.222189):0.142305,Halichondria:0.280013):0.155525):0.
137859);
end;
```

Figure S8. ML tree of fungal and 'double' type TPPPs

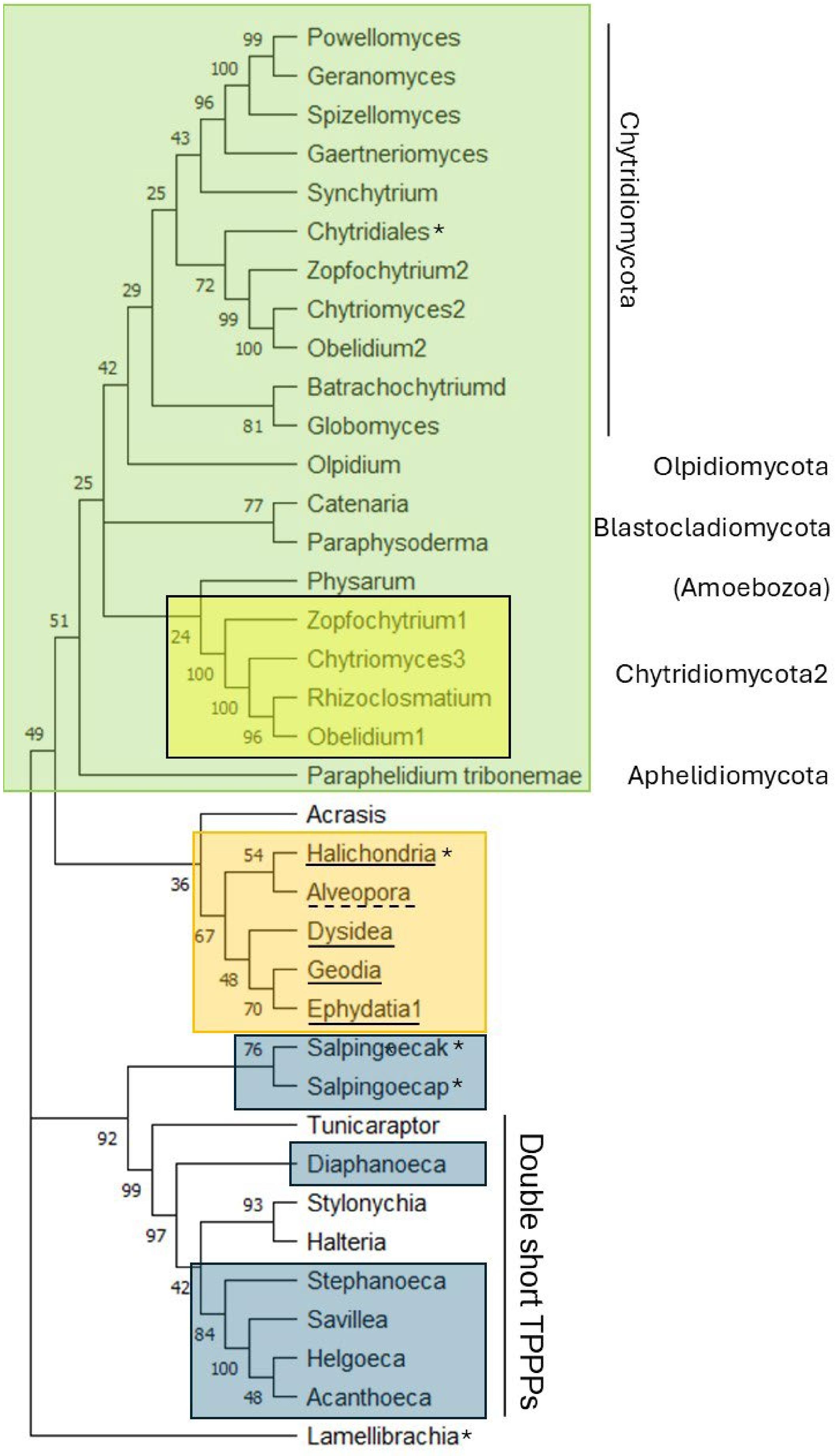

The root of the tree was chosen arbitrarily. The Accession Numbers of proteins/TSAs are listed in Table S2. Bootstrap values (1000 replicates) above 24% are shown at branch nodes. Branches corresponding to partitions reproduced in less than 24% bootstrap replicates are collapsed. Color code and labels are the same as in Fig. 4.

```
((((((((((Powellomyces,Geranomyces)0,9900,Spizellomyces)1,0000,Gaertneriomyces)0,9680,Synch
ytrium)0,4340,(Chytridiales,(Zopfochytrium2,(Chytriomyces2,Obelidium2)1,0000)0,9900)0,7240)0
,2580,(Batrachochytriumd,Globomyces)0,8120)0,2960,Olpidium)0,4240,(Catenaria,Paraphysoderma)
0,7700,(Physarum,(Zopfochytrium1,(Chytriomyces3,(Rhizoclosmatium,Obelidium1)0,9600)1,0000)1,
0000)0,2460)0,2500,Paraphelidium_tribonemae)0,5160,(Acrasis,((Halichondria,Alveopora)0,5480,
(Dysidea,(Geodia,Ephydatia1)0,7020)0,4820)0,6720)0,3680)0,4940,((Salpingoecak,Salpingoecap)0
,7600,(Tunicaraptor,(Diaphanoeca,((Stylonychia,Halteria)0,9340,(Stephanoeca,(Savillea,(Helgo
eca,Acanthoeca)0,4860)1,0000)0,8400)0,4280)0,9700)0,9900)0,9200,Lamellibrachia);
```
